# Quercetin/Zinc complex and stem cells: A new drug therapy to ameliorate glycometabolic control and pulmonary dysfunction in diabetes mellitus: Structural characterization and genetic studies

**DOI:** 10.1371/journal.pone.0246265

**Published:** 2021-03-04

**Authors:** Moamen S. Refat, Reham Z. Hamza, Abdel Majid A. Adam, Hosam A. Saad, Adil A. Gobouri, Fatimah S. Al-Harbi, Fawziah A. Al-Salmi, Tariq Altalhi, Samy M. El-Megharbel

**Affiliations:** 1 Department of Chemistry, College of Sciences, Taif University, Taif, Saudi Arabia; 2 Department of Chemistry, Faculty of Science, Port Said University, Port Said, Egypt; 3 Biology Department, Faculty of Science, Taif University, Taif, Saudi Arabia; 4 Zoology Department, Faculty of Science, Zagazig University, Zagazig, Egypt; 5 Department of Chemistry, Faculty of Science, Zagazig University, Zagazig, Egypt; Children’s Hospital Boston, UNITED STATES

## Abstract

Medicinal uses and applications of metals and their complexes are of increasing clinical and commercial importance. The ligation behavior of quercetin (Q), which is a flavonoid, and its Zn (II) (Q/Zn) complex were studied and characterized based on elemental analysis, molar conductance, Fourier-transform infrared (FTIR) spectra, electronic spectra, proton nuclear magnetic resonance (^1^H-NMR), thermogravimetric analysis, and transmission electron microscopy (TEM). FTIR spectral data revealed that Q acts as a bidentate ligand (chelating ligand) through carbonyl C(4) = O oxygen and phenolic C(3)–OH oxygen in conjugation with Zn. Electronic, FTIR, and ^1^H-NMR spectral data revealed that the Q/Zn complex has a distorted octahedral geometry, with the following chemical formula: [Zn(Q)(NO_3_)(H_2_O)_2_].5H_2_O. Diabetes was induced by streptozotocin (STZ) injection. A total of 70 male albino rats were divided into seven groups: control, diabetic untreated group and diabetic groups treated with either MSCs and/or Q and/or Q/Zn or their combination. Serum insulin, glucose, C-peptide, glycosylated hemoglobin, lipid profile, and enzymatic and non-enzymatic antioxidant levels were determined. Pancreatic and lung histology and TEM for pancreatic tissues in addition to gene expression of both SOD and CAT in pulmonary tissues were evaluated. MSCs in combination with Q/Zn therapy exhibited potent protective effects against STZ induced hyperglycemia and suppressed oxidative stress, genotoxicity, glycometabolic disturbances, and structural alterations. Engrafted MSCs were found inside pancreatic tissue at the end of the experiment. In conclusion, Q/Zn with MSC therapy produced a synergistic effect against oxidative stress and genotoxicity and can be considered potential ameliorative therapy against diabetes with pulmonary dysfunction, which may benefit against COVID-19.

## Introduction

Type 2 diabetes mellitus is highly prevalent, becoming an important global public health concern, particularly in recent times. Diabetes mellitus is a metabolic disease likely caused by defective insulin secretion and more likely by oxidative injury and dysfunction of β cells [1]. Inflammation adversely affects insulin sensitivity and worsens diabetic complications, such as vasculopathy and pulmopathy [2]. With the emergence of an increasing number of silent killer viruses such as coronavirus 2 (SARS-coV-2) that duplicated diabetes mellitus risk especially in patients with pulmonary dysfunction. World Health Organization (WHO) reported in March 2020, SARS-coV-2 is the underlying pathogen of the coronavirus disease-19 (COVID-19) pandemic. This disease is characterized by the primary symptoms of fever and severe pneumonia [3] and represents a serious threat to patients with diabetes. Therefore, Diabetics are more vulnerable to this disease, which is a crucial health issue [4]. Therefore, highly effective therapies that can improve insulin sensitivity; promote pancreatic β cell survival, regeneration, and functions; and enhance immunity would be beneficial to this patient population, specifically in the current time.

Mesenchymal stem cells are a population of self-renewable mature cells that can be harvested from multiple tissues, including bone marrow and adipose tissues [5]. In animal studies, injection of bone marrow-derived mesenchymal stem cells (MSCs) improved the insulin sensitivity of a rodent model of diabetes mellitus [6]. In human clinical trials, injection of bone marrow-derived MSCs to patients with diabetes, specifically those with type 2 diabetes mellitus, improved the function of β cells, reduced the incidence of diabetic complications, and even led to insulin independence in some patients [7].

Quercetin (Q) is a naturally occurring flavonoid that is widely distributed in plants and natural foods and shows many beneficial effects such as anti-inflammatory, antioxidant, and anti-diabetic activities [8]. Additionally, Q administration extended the lifespan of some experimental model organisms and minimized oxidative injury [9]. Q supplementation plays important roles in normalizing blood glucose levels, reducing serum cholesterol and enzyme levels. Moreover, Q improves antioxidant potential and prevents oxidative damage, which are important issues associated with diabetes. In an experimental rat model of diabetes, Q exerted protective effects through regulating inflammatory responses (particularly those resulting from viral infection), promoting pancreatic islet regeneration, and increasing insulin release [10].

Beneficial effects of various nutrients in promoting the immune response of patients with diabetes have garnered much attention. In particular, the role of micronutrients, such as Zn, in the treatment of diabetes mellitus have been extensively studied [11]. Zn dysregulation occurs in both types of diabetes. A previous studies have proposed a link between Zn level alterations and pancreatic β cell dysfunction based on the association between the risk of type 2 diabetes mellitus and polymorphisms of the Zn transporter gene *ZnT8* [12]. Additionally, Zn plays vital roles in the treatment of pulmonary diseases [13]. In a previous meta-analysis, six studies performed in India, Peru, South Africa, and Bangladesh indicated that Zn supplementation inhibited pneumonia, but low dietary Zn intake practically decreased resistance against infections [14]. The presence of diabetes led to lung growth retardation in both young and adult rats, and lung distensibility was decreased in young rats. Elevated levels of specific connective tissue proteins may have led to lung distensibility [15].

**Fig 1:**
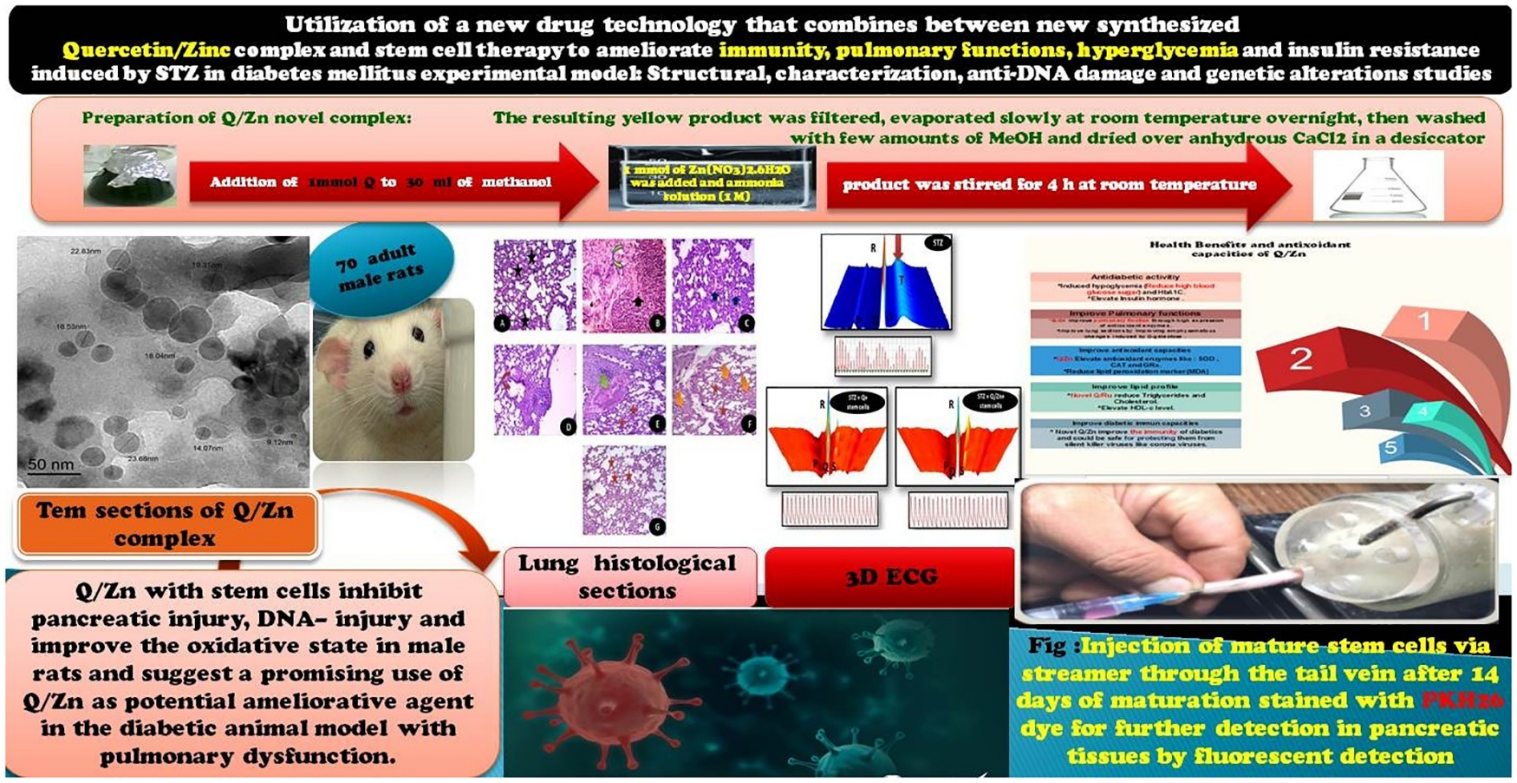
Graphical abstract.

So, our current study was designed as a mimic to diabetes mellitus experimental model with pulmonary dysfunction and then treatment by combination of MSCs and novel complex by chelating between Q and Zn (Q/Zn). Our proposed therapy explored the action of a novel (Q/Zn) complex in alleviating a series of diabetes mellitus complications and succeeded in alleviating hyperglycemia and glycometabolic disturbances and restored normal biochemical parameters and normal pancreatic and pulmonary structures with potent antioxidant activities as shown in graphical abstract (Fig 1), which will be a suggested protective therapy against COVID-19 and any inflammatory diseases.

## Materials and methods

### Chemicals and analyses

All chemicals (quercetin and zinc(II) chloride hexahydrate) were purchased from Sigma-Aldrich and used without purification. Zinc(II) complex with Q were synthesized by the following method. The analyses and the corresponding models are summarized in Table 1.

**Table 1:** Instrumental analysis and the corresponding models.

*Type of analysis*	*Models*
Elemental analyses	Perkin Elmer CHN 2400
Conductance	Jenway 4010 conductivity meter
FTIR spectra	Bruker FTIR Spectrophotometer
^1^HNMR spectra	Varian Mercury VX-300 NMR spectrometer, 300 MHz
Electronic spectra	UV2 Unicam UV/Vis Spectrophotometer
Thermo gravimetric	TG/DTG–50H, Shimadzu thermo-gravimetric analyzer
TEM	JEOL 100s microscopy

### Synthesis of new Q/Zn complex

To the 1 mmol of Q dissolved in methanol (30 mL) 1 mmol of Zn(NO_3_)_2_.6H_2_O was added and ammonia solution (1 M) at pH = ~ 8. Then, the product was stirred for 4 h at room temperature. After stirring, the resulting yellow product was filtered, evaporated slowly at room temperature overnight, then washed with few amounts of MeOH and dried over anhydrous CaCl_2_ in a desiccator.

### Experimental animals

Seventy adult male albino rats weighing 170–200 g were maintained in metal cages under controlled, specific pathogen-free conditions, ensuring good ventilation. The animals were provided healthy diets. During experiments, all efforts were made to alleviate animal suffering. The experimental protocol (Fig 1) was approved by the Zagazig University Ethical Committee (approval number: ZU-IACUC/1/F/41 I2018) and followed international guidelines for animal use and care.

### Isolation and culture of bone marrow MSC

MSCs were isolated from bone marrow as described by Wang et al. [16]. Briefly, bone marrow was harvested from the tibias and femurs of 10 rats, and the harvested cells were isolated by separating mononuclear cells using the Ficoll–Hypaque gradient. These cells were allogeneic as these cells are immunologically inert, so it cannot be identified by host immune cells. Cells were cultured in Dulbecco’s modified Eagle’s medium (DMEM) with fetal bovine serum (FBS) for 3 h at 37°C in a CO_2_ incubator. Non-adherent cells were carefully removed after 3 h, and the medium was replaced with fresh one. The medium was changed every 48 h and replenished with DMEM with 10% FBS. At 80% confluency, cells were detached using trypsin–EDTA. Purified MSC populations were obtained from near-confluent cultures.

### Flow cytometry

Bone marrow-derived MSCs were analyzed using flow cytometry (Becton Dickinson). A total of 0.4 × 10^6^ MSCs were incubated with specific individual monoclonal antibodies, conjugated with fluorescence isothiocyanate (FITC) and phycoerythrin (PE) in 250 μl phosphate buffered saline for 30 min in the dark at room temperature. The following cell surface antigens were observed CD29-FITC, CD34-FITC and CD105-PE. CD 29 is known as stromal precursor of bone marrow and highly expressed in MSCs [17, 18]. CD105 (endoglin) is expressed in MSCs and hematopoietic stem cells. CD 34 is cell surface marker widely used in isolation and identification of hematopoietic stem and progenitor cells. They are expressed exclusively on hematopoietic stem cells [19] Mouse isotype-matched IgG served as a negative control (BD Pharmingen). 100,000 labeled cells were acquired and analyzed using CellQuest software [20].

### Experimental induction of diabetes mellitus

After acclimatization period, male albino rats (body weight, 170–200 g) were divided into seven groups (10 rats per group). Group I (control group) was treated with physiological saline (i.p.); group II (STZ group) received a single dose (i.p.) of STZ (50 mg·kg^-1^) [21]; group III (diabetic rats) underwent MSCs therapy (The diabetic rats were injected (i.v) with MSCs derived from bone marrow (2x10^6^ per rat) through the tail vein; group IV (diabetic rats) received a single dose (i.p) of Q (30 mg·kg^-1^) [22]; group V (diabetic rats) was treated with the novel Q/Zn complex (i.p.) at the same dose described previously; groups VI was exposed to STZ and then received MSCs therapy plus Q daily (i.p.); and group VII was exposed to STZ and then received MSCs therapy plus Q/Zn.

Experimentally, diabetes mellitus was induced by using a freshly prepared STZ solution (STZ dissolved in an acidified buffer solution, PH = 4.0) administered to fasted animals via injection (i.p.) within a few minutes after preparation (50 mg·kg^-1^) [21]. The animals were fed with 10% sucrose in place of water on the 1^st^ day of injection to avoid severe hypoglycemia, Sucrose was removed and replaced with fresh water in the next day. The treatments were started after 72 h of diabetes induction and continued daily for 30 consecutive days. The diabetic status was evaluated by measuring blood glucose levels after 3 days of STZ injection. Blood glucose levels above 280 mg·dL^-1^ were indicative of diabetes. We made 2^nd^ measurement after another 72 h to confirm unrecovered of glycaemia and then begin treatments and we made continuous checkup of body weight and blood glucose at each 1^st^ day of each week of the experimental period and as needed to check the glycemic status.

### Blood collection

Under light anesthesia, blood samples from the eye plexus were collected in capillary tubes. The samples were centrifuged at 6,000 rpm for 15 min for biochemical analyses. The animals were decapitated using ethical practices, and the pancreatic tissues were collected and stored at -25°C for further investigations.

### Determination of the blood glucose level

Levels of the blood glucose were evaluated by using commercial kits of Biodiagnostic co.

### Measurements of serum insulin, C-peptide, and HbA1c

Fasting serum insulin levels were evaluated using a rat ELISA kit (ALPCO Diagnostics, USA). C-peptide (Sigma-Aldrich) and HbA1c (Cusabio Co., China) levels were measured using respective ELISA kits according to the manufacturers’ protocols.

### Lipid biomarkers determination

Serum total cholesterol (TC) and triglyceride (TG) levels were determined using the methodologies described by Carr et al. [23] and Warnick et al. [24], and LDL-c and v-LDL-c levels were calculated.

### Preparation of pancreatic tissue homogenates

A small portion of the pancreatic tissues was used for estimating antioxidant biomarkers. Pancreatic tissues were homogenized in (5 mL) cold buffer and centrifuged at 5,000 rpm for 1–2 h. The obtained supernatant was stored at -20°C.

### Determination of oxidative stress markers

The supernatants of pancreatic tissue homogenates were used for estimating myloperoxidase (MPO) and xanthine oxidase (XO) levels. The MPO Kit (ab111749) is a rapid and practical method of MPO detection [25]. XO level was determined using the method described by Litwack et al. [26]. Reduced glutathione level was determined using the method described by Sedlak and Lindsay [27]. Pancreatic malondialdehyde (MDA) content was determined using the method described by Ohkawa et al. [28]. Catalase (CAT) level was determined using the method described by Beers and Sizer [29].

### Illustrative 3D parametric ECG (three-dimensional ECG)

Male rats in the treated groups (STZ, STZ + Q and stem cells, and STZ + Q/Zn and stem cells) were subjected to light anesthesia using thiobarbital. Then, saline was injected in the jugular vein, and heart beats were estimated using ECG, recorded using LabChart, and illustrated.

### Transmission electron microscopic study (TEM)

For ultrastructural examination, Pancreas portion was fixed in 2.5% glutaraldehyde for 48 h. The pancreatic specimens were washed in (PB) "phosphate buffer solution" (pH 7.4) for 4 times / 20 min for each time and then post-fixed in a buffered solution of "1% osmium tetraoxide" for about 2 h. Fixed pancreatic specimens were dehydrated in ascending grades of ethyl alcohol (30, 50, 70, 90, and 100%), then cleared in 2 changes of "propylene oxide", and embedded in Epon resin (Hayat. 1986 [30]).

Semi-thin sections (1 μm thick) were stained with toluidine blue for 2 min and examined by using a light microscope. Ultrathin sections (60–90 nm thick) and representative fields of semithin sections were selected and were cut with a diamond knife using a Reichert OMVs ultramicrotome, mounted on copper grids and then double stained with uranyl acetate and lead citrate. The grids were examined by using a transmission electron microscope (JEOL JEM-1200 EX II, Japan) operated at 60–70 kV, Faculty of Agriculture (Electron microscope unit), Mansoura University.

### Single cell gel electrophoresis (SCGE) (Comet assay)

Pancreatic tissues were placed in a Petri dish containing a prechilled lysis solution (with Ca^2+^and Mg^2+^ but without EDTA), and cell viability was determined. Comet assay was performed under alkaline conditions, as described by Singh et al. [31], with some modifications. In brief, tissue slides were covered with a layer of 0.5% normal agarose. Lymphocytes were isolated and washed with a washing buffer. Then, 50 μL aliquots of the cell suspension were mixed with 100 μL of 0.5% low melting agarose, placed on slides, and immediately covered with coverslips. After removing the coverslips, all slides were immersed in a lysis solution (2.5 M NaCl, 100 mM EDTA, 10 mM Tris, NaOH to pH 10, 1% *N*-lauryl sarcosine, 1% Triton X-100, and 10% DMSO) and incubated for 1 h at 4°C in the dark. The slides were then placed in an electrophoresis tank containing freshly prepared alkaline buffer (300 mM NaOH and 1 mM EDTA; pH > 13), and electrophoresis was conducted at temperature 37° C for 20 min at 300 mA and 25 V. After electrophoresis, the slides were washed three times first with a neutralizing buffer (0.4 M Tris, pH 7.5) for 5 min each and then with ethanol for the same time for fixation. Finally, DNA was stained with 60 μL of ethidium bromide (20 μL·mL^-1^). Two slides were prepared for each sample, and 50 cells were randomly selected for image analysis under a fluorescence microscope (Leica). All results were evaluated in terms of nine image analysis parameters. All examinations were performed at the clinical pathology unit of the National Cancer Institute Kasr El-Einy, Faculty of Medicine, Cairo University.

### Histological analysis of pancreas and lung tissues

Pancreatic and lung tissues were fixed in 10% neutral-buffered formalin. Following fixation, the samples were embedded in paraffin, sectioned, stained with hematoxylin and eosin, and examined under a light microscope.

### Semi-quantitative Ploymerase Chain Reaction (PCR)

PCR amplification of lung SOD and CAT was performed using specific pair of primers (synthesized by Macrogen Co., GAsa-dong, Geumcheon-gu., Korea) for rat. The sequences of specific primers and product sizes are listed in Table 2.

**Table 2:** Primers and PCR conditions used for the tested genes.

Gene name (Abbreviation); Accession number	Primer sequence (5’–3’)	Product size
Mouse Catalase (mCAT); NM_009804.2	Forward–GCCAATGGCAATTACCCGTC	532 bp
	Reverse–AGAATGTCCGCACCTGAGTG	
Mouse Superoxide dismutases (mSOD); BC066063.1	Forward–GGAGAGCAGCGGTCGT	631 bp
	Reverse–TGTGGTATTGGAGGTTGGGTC	
Mouse Beta-actin (mΒ-actin); NM_007393	Forward–TATAAAACCCGGCGGCGCA	516 bp
	Reverse–ATGGCTACGTACATGGCTGG	

4 microliters of cDNA and 1.25 μM of each primer were added to 12.5μM 2xGoTaq PCR master mix (Promega Corporation, Madison, WI, USA). The volume was completed to 25 μl with DNase free water and samples were loaded into Techne TC-3000x thermal Cycler (Bibby Scientific, England). PCR conditions were 94 C for 5 mins followed by cycles of 1 min at 95°C, 1 min at annealing temperature 60°C and 1 min at 72°C. The cycle number was adjusted so that all reactions were within the linear range of product amplification according to the reference gene (28 cycles). The final extension step was 7 min at 72°C, PCR products were separated on 1.5% agarose-A (Bio Basic, Markham, ON, Canada) gel in 1.0 X-TAE (Tris–Acetate-EDTA) buffer (Sigma–Aldrich, St. Louis, MO, USA) at 100 V for 30 min.

The gel was stained with ethidium bromide (Sigma–Aldrich), visualized and photographed under UV light using Ingenius gel documentation system (Syngene Europe, Cambridge, UK) [20].

### Statistical analysis

Statistical analyses were performed using SPSS 17.0 for Windows. Data are presented as mean and standard error. Differences among groups were determined using one-way ANOVA, followed by least square difference post hoc test, using SPSS 17.0. Results were considered significant at P-value ≤ 0.05, considered highly significant at P-value ˂ 0.001, considered non-significant P-value ˃ 0.05 [32].

## Results

### Chemical interpretations of zinc(II) quercetin complex (Q/Zn)

#### Elemental analysis and conductance measurements

**Fig 2:**
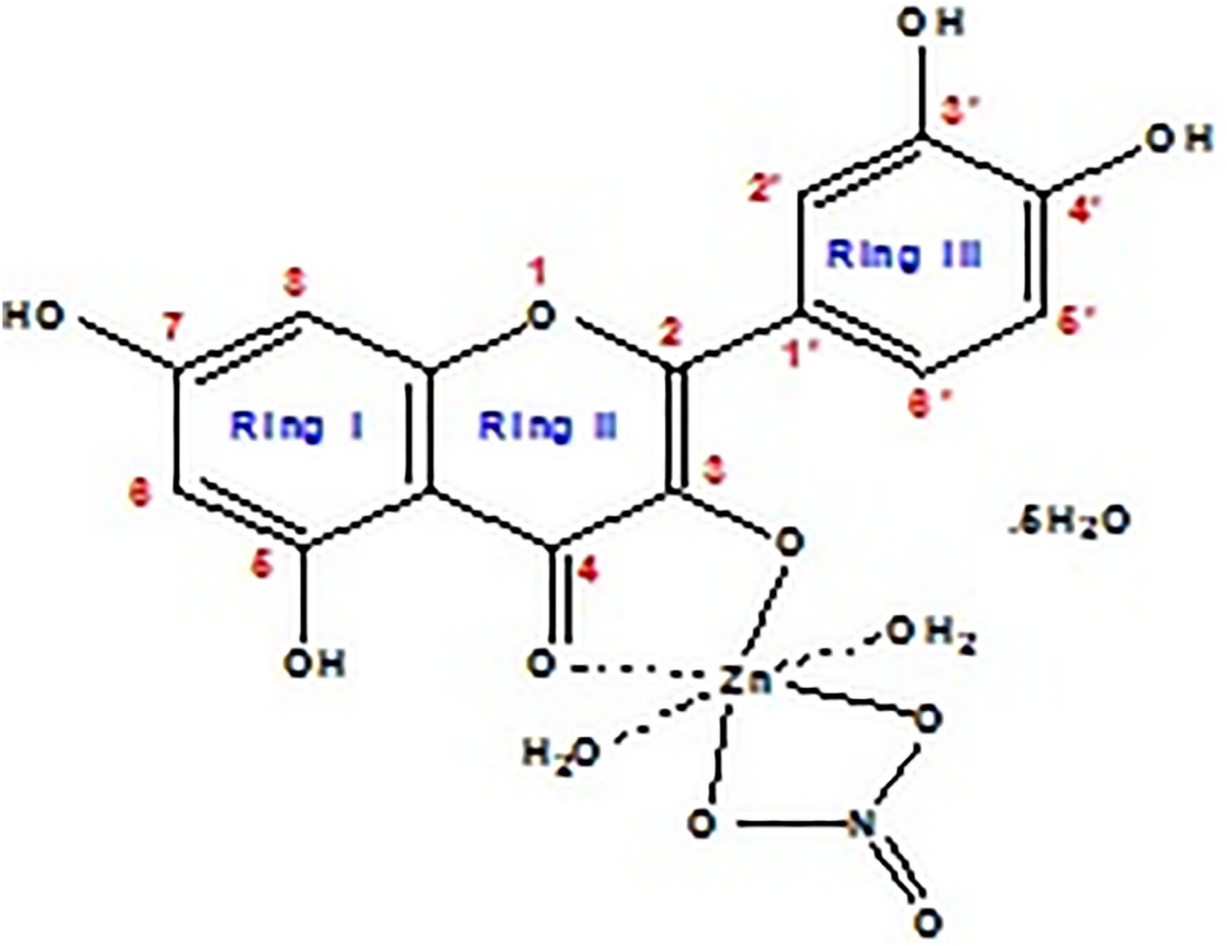
Speculated structure of Q/Zn complex.

Micro analytical analysis was done to support the composition and proposed structure of the Q/Zn complex (Fig 2). This new Q/Zn complex was stable in air and soluble in DMSO and DMF solvents. The molar conductivity (Λ_m_) value of Q/Zn complex was 15 ohm^-1^.cm^2^.mol^-1^, that considered as non-electrolytes nature [33].

We try to collect a single crystal in pure form but didn’t succeed. The composition of synthesized zinc (II) complex was assigned according to elemental analysis as follows: MF, C_15_H_23_NO_17_Zn; Mol. Wt.: 554.73 g/mol; Calculated (%), C, 32.48; H, 4.18; N, 2.52; Zn, 11.79. Experimental data (%), C, 32.13; H, 4.09; N, 2.45; Zn, 11.37. The yield of the solid zinc(II) complex was about 75%. The solid product has a higher melting point 2>50 °C.

#### Infrared spectra

**Fig 3:**
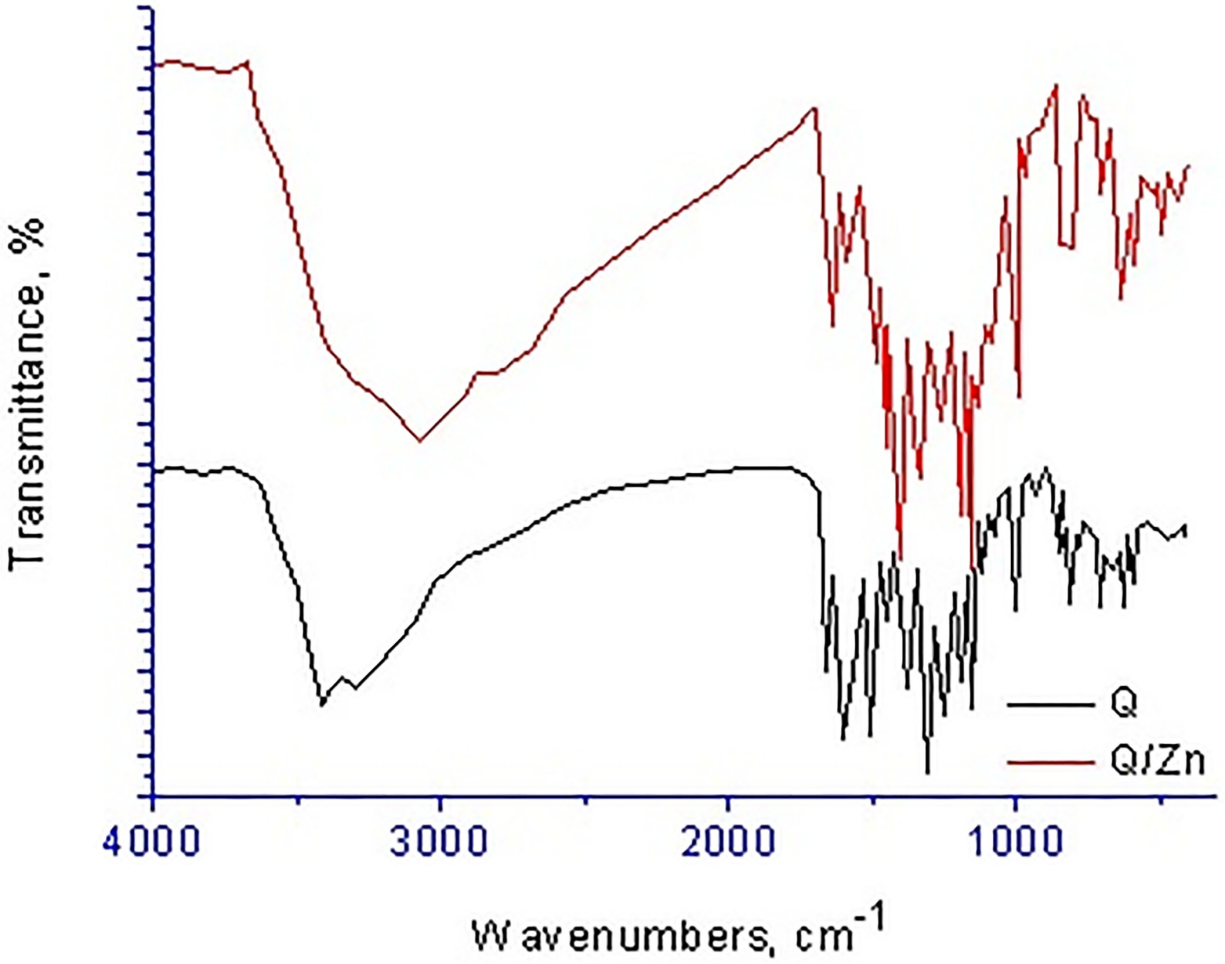
Infrared spectra of free Q ligand and its zinc(II) complex.

Infrared spectra of both quercetin (Q) free chelate and its zinc(II) complex (Q/Zn) are scanned and displayed in Fig 3, the assignments of their frequencies can be summarized as follows; for free Q ligand, the broad strong bands presence at 3416 and 3301 cm^-1^ attributed to stretching frequencies ν(O–H) of–OH of polyphone groups, also in case of Q/Zn complex the very strong broad band at 3397 cm^-1^ is attributed to the presence of coordinated or uncoordinated water molecules. This detected vibrations are agreement with the suggested structure (Fig 2) of Q/Zn complex. The stretching vibration band of carbonyl group ν(C4 = O) in case of the Q ligand take place at 1661 cm^-1^, while the Q/Zn complexity this band is presence at 1643 cm^-1^. This result revealed that the Zn(II) metal ion can be coordinated through the carbonyl oxygen atom C(4) = O and C(3)–OH or C(5)–OH group of the Q chelate [34]. Regarding Q free ligand, the stretching vibration band ν(COC) of ether group in the ring II is existed at 1253 cm^-1^, this band did not shifted after complexation due to there is not involved in the coordination. The vibration band of ν(C–OH) occurs at 1386 cm^-1^ for the Q free ligand, this peak is shifted to 1358 cm^-1^ for Q/Zn complex because of involvement of the oxygen phenolic group C(3)–OH (ring II) on the coordination.

The distinguish vibration bands of the bidentate NO_3_^-^ group, are observed at 1320 and 1111 cm^-1^ due to ν_as_(NO_2_); A’’ and asymmetric stretching vibration of (NO_2_); A’, respectively [34]. The frequency (ν(N = O)) is appeared at 1481 cm^-1^ as a medium band, while the two bending motion of the type δ(NO_2_); A’’ are appeared at 752 and 703 cm^-1^ as medium-to-weak bands. The presence of other bands at 646 and 532 cm^-1^ are attributed to stretching vibration ν(Zn–O), this confirm the formation of metal complex [35].

#### UV–Vis electronic spectra

**Fig 4:**
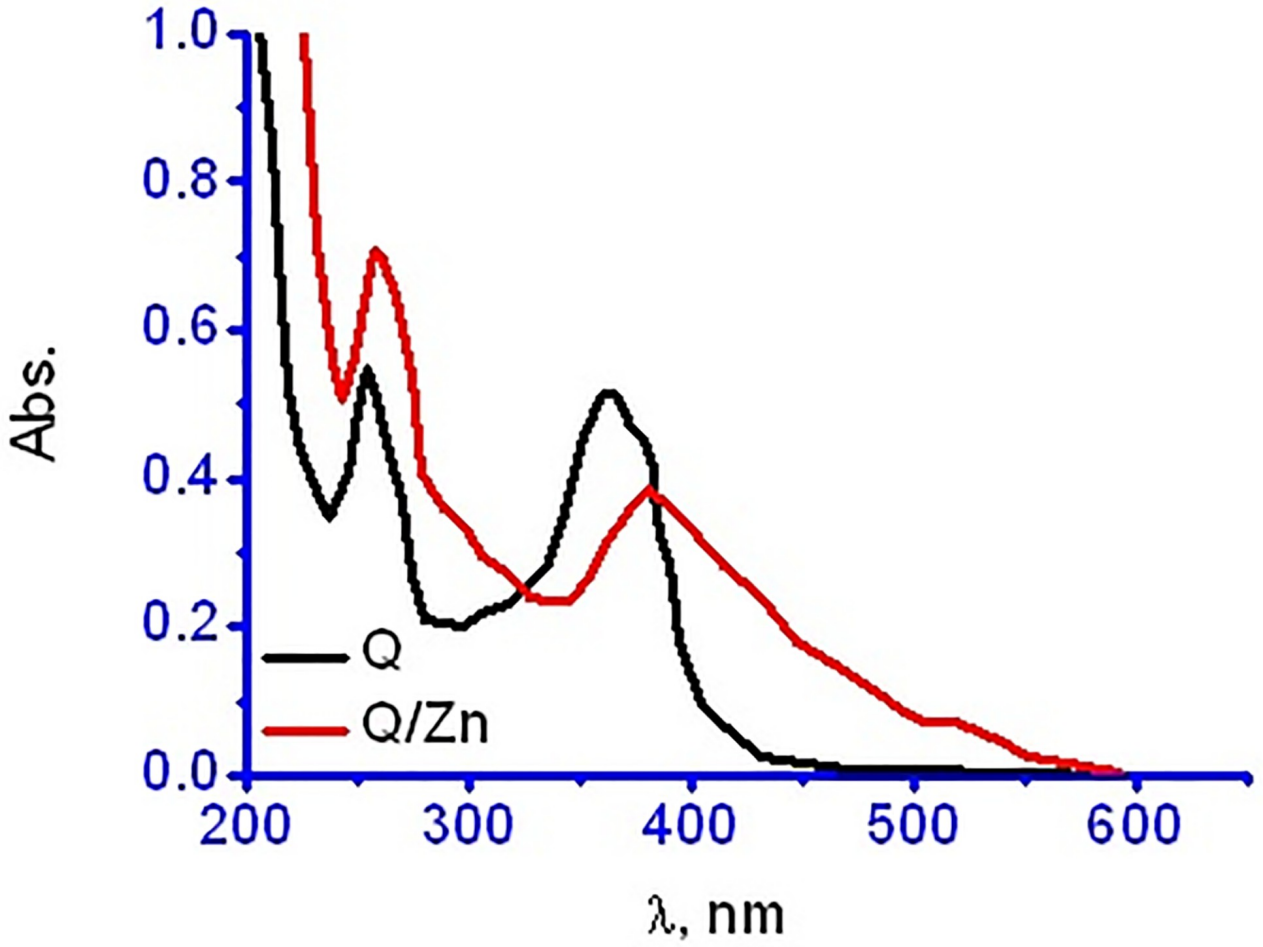
UV-Vis spectra of free Q ligand and its zinc(II) complex.

The UV-Vis electronic data of quercetin and its zinc(II) complexity were scanned and shown in Fig 4. The electronic spectrum of Q ligand has a two distinguish bands at 254 and 363 nm due to n→π* and π→π* electronic transitions of conjugated system between (ring I and carbonyl of ring II) and (carbonyl of ring II and ring III) respectively. In comparison between Q and Q/Zn complex, the electronic spectrum of Q/Zn complex show that both two observed bands are bathochromic shifted to 258 and 381 nm because of the complex formation. This bathochromic shift may be due to the extension of the conjugated system with the complexation [36].

#### ^1^H-NMR spectra

**Fig 5:**
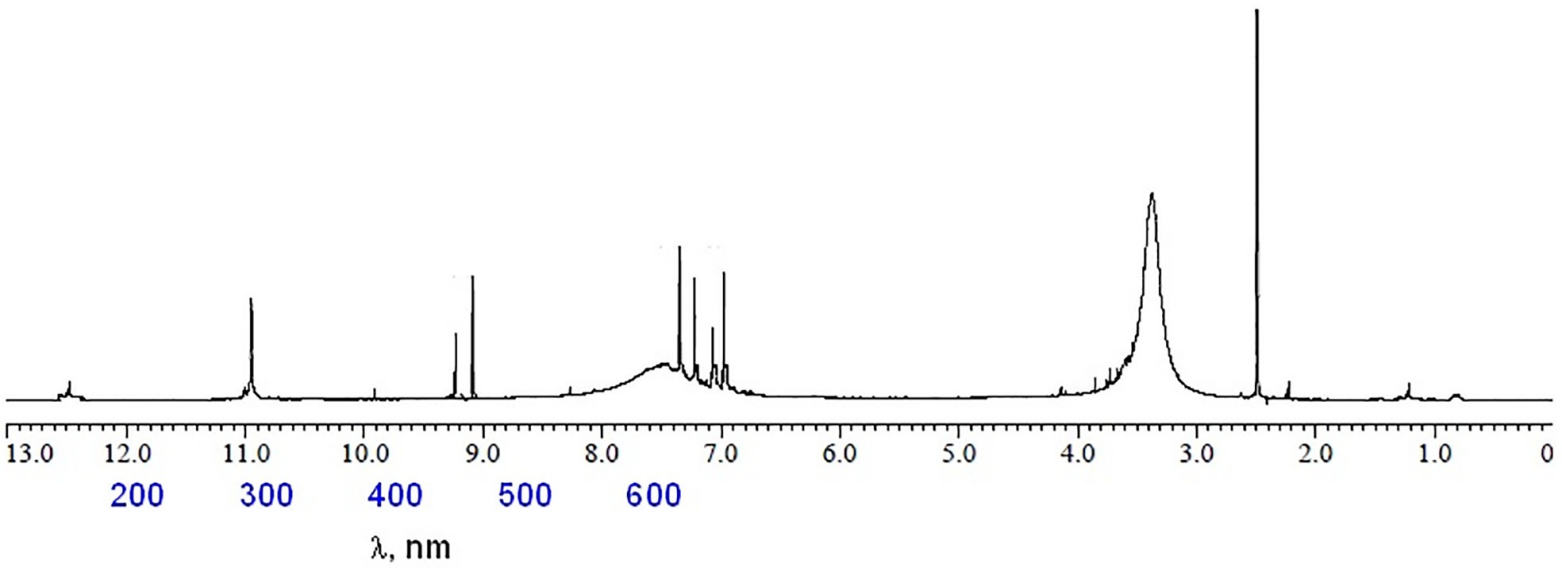
^1^H-NMR spectrum of zinc(II) complex.

^1^H-NMR spectrum of free Q ligand (400 MHz, DMSO-d_6_): δ 12.48 (1H, 5-OH), 10.77 (1H, 7-OH), 9.58 (1H, 3-OH), 9.39 (1H, 4′-OH), 9.31 (1H, 3′-OH), 7.64 (1H, 2′-H), 7.53 (1H, 6′-H) 6.88 (1H, 5′-H), 6.39 (1H, 8-H), 6.13 (1H, 6-H) [37]. ^1^H-NMR spectrum of [Zn(Q)(NO_3_)(H_2_O)_2_].5H_2_O complex (Fig 5): (400 MHz, DMSO-d_6_): δ 12.51 (1H,5-OH), 10.95 (1H, 7-OH), 9.35 (1H, 4′-OH), 9.28 (1H, 3′-OH), 7.46 (1H, 2′-H), 7.34 (1H, 6′-H), 7.15 (1H, 5′-H), 6.93 (1H, 8-H), 6.72 (1H, 6-H). From the chemical shift data of the Q/Zn complex, it can be show the absent of δ 9.58 (1H, 3-OH) group proton which esxisted in Q free ligand. However, the protons of 7-OH, 5-OH, 3´-OH and 4´-OH groups were did not affected after complexation. The strong broad signal at δ 3.45 ppm that present in case of Q/Zn complex is refered to the coordinated protons and uncoordinated water molecules. These results confirmed the place of coordination through carbonyl C(4) = O oxygen and phenolic C(3)–OH oxygen.

#### Thermogravimetric analysis

**Fig 6:**
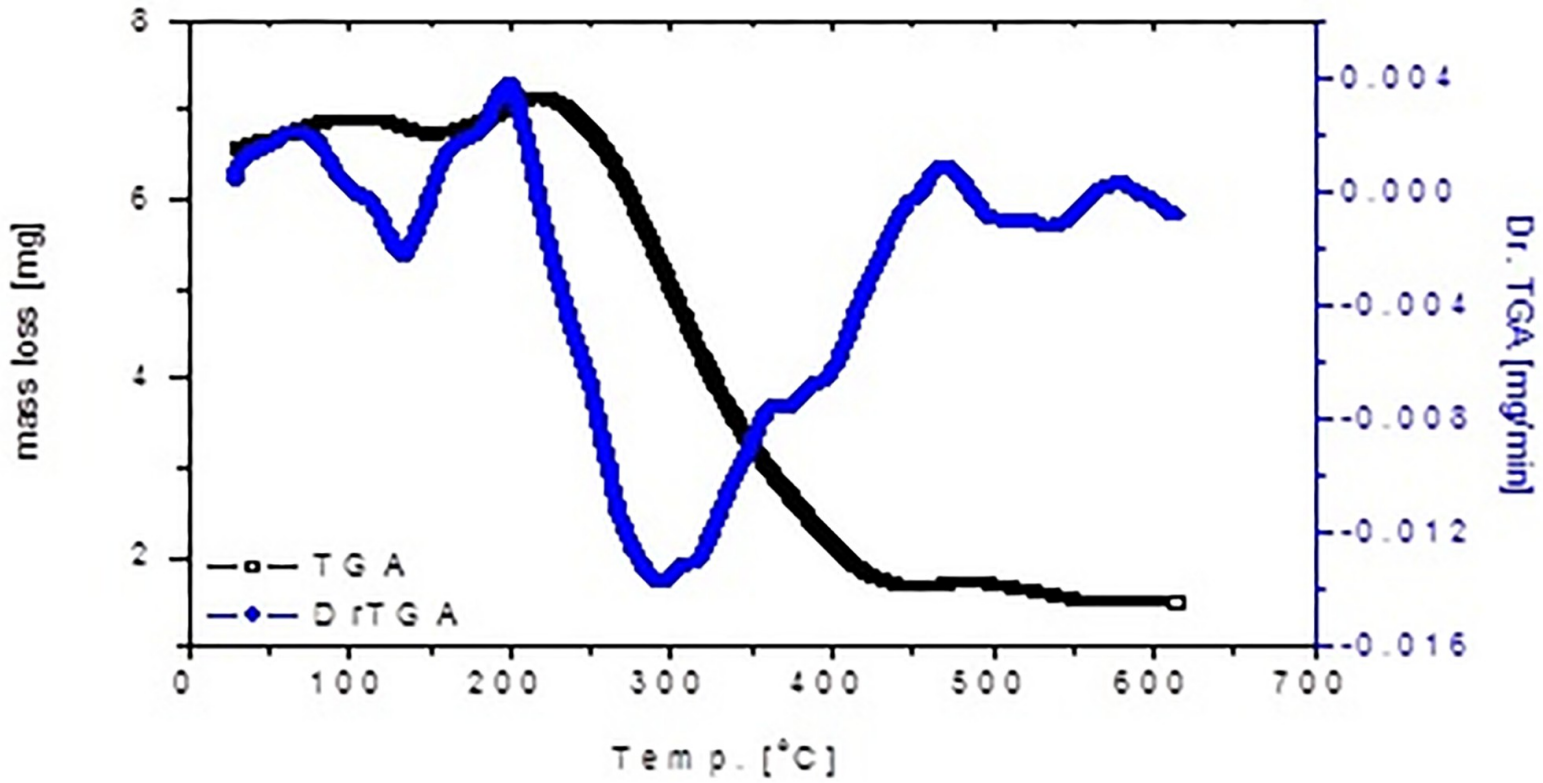
TGA-DrTGA curves of zinc(II).

The thermogravimetric (TGA) and its differential (DrTGA) analysis of [Zn(Q)(NO_3_)(H_2_O)_2_].5H_2_O complex were performed in the temperature range of 30–600°C under nitrogen atmosphere (Fig 6). The first endothermic decomposition step at DrTGA = 130°C is attributed to remove one crystalline water molecules. The second thermal craking step is inserted between 130–450°C at DrTGA = 290 °C corresponded to release of uncoordinated 4H_2_O, coordinated 2H_2_O, NO_2_ gas, and quercetin ligand molecules. The following third endothermic DrTGA stage at 520 °C, which doesn’t accompanied by any mass loss due to solid-solid interaction and change in physical behavior, while the final solid residual product is concluded to be ZnO.

### TEM examination of zinc (II) quercetin complex

**Fig 7:**
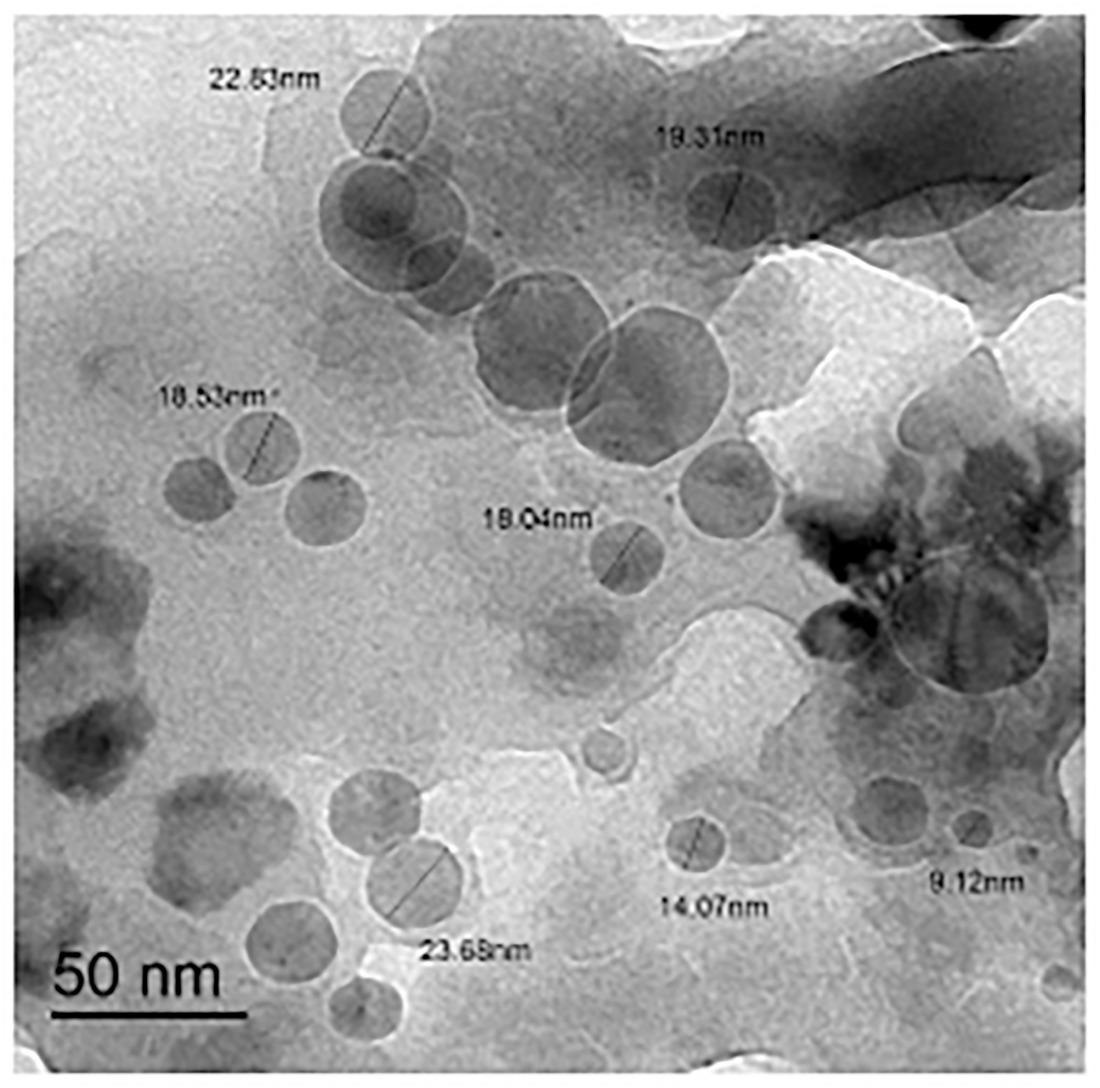
TEM image of zinc(II) complex.

Fig 7 shows the transmission electron microscopy (TEM) photograph of the synthesized zinc (II) quercetin (Q/Zn) complex. The uniform matrix of the synthesized complex in the pictograph was clear, confirming that the Q/Zn (II) complex has a homogeneous material phase. Black, spherical spots, with a particle size of 9–22 nm were observed in the Zn (II) complex.

### Biological results of zinc(II) quercetin complex (Q/Zn)

#### Characterization of MSCs

**Fig 8:**
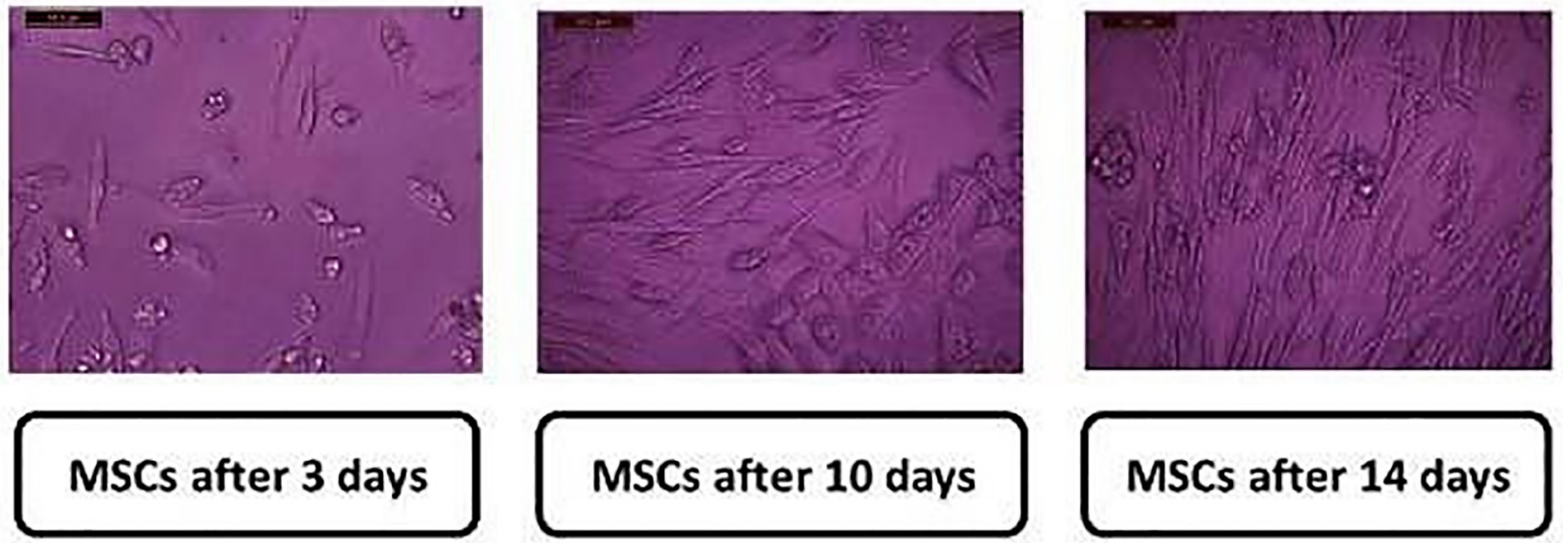
Characterization of isolated bone marrow stem cell of rat which was used after 14 days (mature stage) X200.

The isolated stem cells appeared clearly as after 3, 10, and 14 d of isolation. MSCs were identified by their spindle shape and fibroblast- like morphology (Fig 8) and detected using the stem cell markers CD34, CD29, and CD105. MSCs were positive for CD29 (>98%), CD105 (96.5% ± 1.54), and CD34 (0.04% ± 0.01%). The cells appeared healthy and were prepared for injection into the tail vein of rats.

#### Immunophenotype characterization of MSCs by using flow cytometry

**Fig 9:**
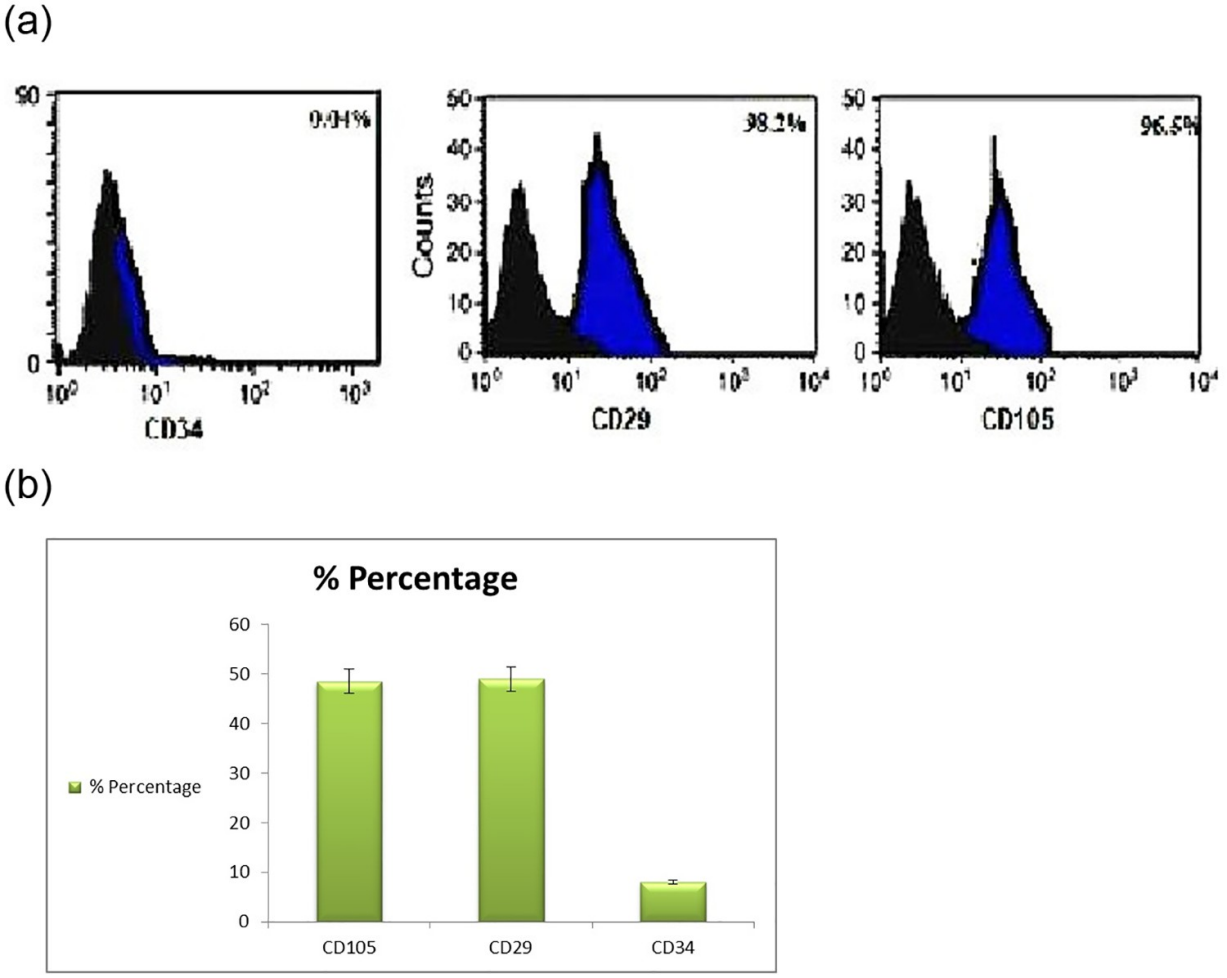
Flow cytometry results of the mesenchymal stem cell markers CD34, CD29, and CD105. MSCs highly expressed CD29 and CD105 (>95%) and weakly expressed CD34 (<5%). There is a significant difference in CD105, CD 29 and CD 34 expression between bone marrow derived mesenchymal stem cells (P < 0.001). The black area represents IgG (negative control) expression, and the blue areas depict marker expression. The results are representative of three independent experiments. Data are presented as mean ± S.D.

Fig 9 presents the Flow cytometry results of mesenchymal stem cells (MSC) markers of CD29, CD105 and CD34 in vitro. MSCs were positively expressed for CD29 (98.2% ± 1.6%), and CD105 (96.5% ± 1.54) while weakly and negatively expressed negative for hematopoietic lineage marker CD34 (0.04% ± 0.01%). The black area represents isotype control IgG expression and blue area represent the marker expression. The results are representative of four independent experiments. Data results correspond to ±standard deviation.

#### Lipid profile

Table 3 shows the lipid profile parameters of rats treated with MSCs, Q and Q/Zn, or both. TG levels increased by 3.09-fold in diabetic rats compared to the normal control group. TG levels decreased by 39.6% and 43.2% in diabetic animals treated with Q and Q/Zn, respectively, as compared to that in diabetic animals. However, in diabetic rats treated with both MSCs and Q/Zn, TG level decreased by 61.91%, compared with the diabetic non-treated group.

**Table 3:** Lipid profile picture of control and treated male rats with quercetin, quercetin/Zn, or stem cells and their combinations.

Groups	Parameters				
	Triglycerides (mg/dl)	Total Cholesterol (mg/dl)	HDL-c (mg/dl)	LDL-c (mg/dl)	vLDL-c (mg/dl)
Control group	61.02±2.36^g^	103.02±2.03^g^	40.22±3.25^ab^	21.03±1.02^g^	12.36±1.65^g^
STZ group	250.36±3.54^a^	280.36±3.69^a^	23.36±2.02^g^	60.36±3.36^a^	50.07±4.02^a^
STZ plus stem cell group	178.02±4.25^b^	210.02±2.36^b^	30.66±2.58^f^	39.25±2.36^b^	35.60±2.36^b^
STZ plus Quercetin (Q) group	151.25±5.25^c^	180.58±4.25^c^	33.36±2.14^e^	35.25±1.36^c^	30.25±2.36^c^
STZ plus Quercetin/Zn (Q/Zn) group	142.36±4.03^d^	160.36±3.69^d^	35.36±2.69^d^	33.32±2.58^d^	28.41±1.36^d^
STZ plus Quercetin (Q) and stem cells	121.01±3.69^e^	145.36±3.69^e^	37.25±2.55^c^	28.25±2.36^e^	24.02±2.36^e^
STZ plus Quercetin /Zn (Q/Zn) and stem cells	95.36±3.02^f^	125.36±1.69^f^	39.04±2.05^b^	23.02±3.94^f^	19.36±0.69^f^

Means within the same column (mean ± SE) (n = 10) carrying different letters are significant at P ≤ 0.05.

The highest value has the symbol (a) and decreasing in value were assigned alphabetically. (While the same symbols are non-significant to each other’s and the different symbols are significant).

TC levels decreased more in the diabetic group treated with both MSCs and Q/Zn than the diabetic group treated with MSCs or Q alone compared to diabetic non-treated animals (Table 3). HDL-c decreased by 41.91% in diabetic rats compared to controls. The level of HDL-c increased in the following treatment order: STZ + MSC + Q/ZN > STZ + MSC + Q > STZ + Q/Zn > STZ + Q > STZ + MSCs. LDL-c and VLDL-c levels increased almost by 2.85- and 4.05-fold in diabetic animals compared to normal control rats, respectively. Treatment of the diabetic group with both MSCs and Q/Zn decreased LDL-c and VLDL-c levels more than that with other treatments (Table 3).

### Blood glucose level, insulin hormone and fasting C-peptide serum

Blood glucose and HbA1c were elevated in the diabetic group by 3.60- and 2.81-fold compared to that in normal control animals, respectively (Table 4). However, insulin and C-peptide levels significantly decreased in diabetic rats as compared to normal control animals. Treatment of diabetic animals with MSCs in combination with Q/Zn improved all parameters better than either MSCs or Q/Zn.

**Table 4:** Blood glucose level, insulin hormone, HBA1C and fasting serum C-peptide of control and treated male rats with quercetin, quercetin/Zn, or stem cells and their combinations.

Groups	Parameters			
	Blood glucose (mg/dl)	Insulin Hormone (uIU/ml)	HbA1C (mmol/mol)	Fasting serum C-peptide (ng/ml)
Control group	85.01 ±3.25^ef^	25.36±1.25^ab^	3.02±0.65^e^	4.02±0.69^a^
STZ group	391.25 ±4.02^a^	1.40±0.25^d^	8.51±1.36^ab^	0.82±0.05^e^
STZ plus stem cell group	270.36 ±4.03^b^	15.36±2.15^c^	7.02±1.02^b^	1.98±0.87^d^
STZ plus Quercetin (Q) group	83.05 ±5.02^f^	23.69±1.36^b^	3.98±1.25^de^	3.52±0.69^c^
STZ plus Quercetin/Zn (Q/Zn) group	84.05 ±4.25^f^	24.25±2.65^b^	3.05±0.69^de^	3.84±0.63^c^
STZ plus Quercetin (Q) and stem cells	189.36±2.65^c^	24.03±2.15^b^	6.01±0.87^bc^	3.54±0.87^bc^
STZ plus Quercetin /Zn (Q/Zn) and stem cells	137.25±4.36^d^	25.65±2.02^ab^	5.54±0.96^c^	4.00±0.96^a^

Means within the same column (mean ± SE) (n = 10) carrying different letters are significant at P ≤ 0.05. The highest value has the symbol (a) and decreasing in value were assigned alphabetically. (While the same symbols are non-significant to each other’s and the different symbols are significant).

### Oxidative stress enzymatic and non-enzymatic biomarkers

**Fig 10:**
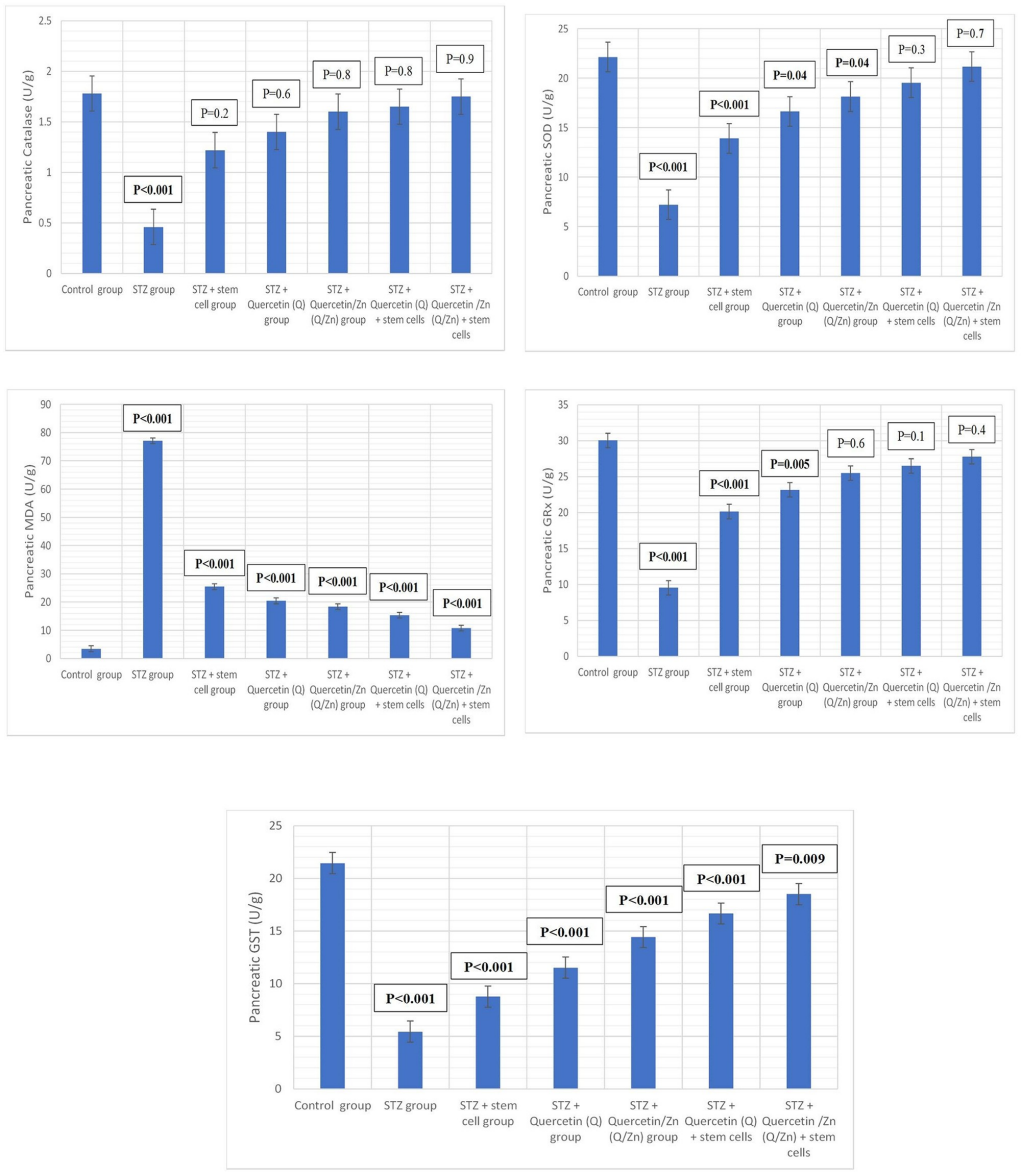
Antioxidant enzymes markers in pancreatic tissues (P value mentioned). Where, P-value ≤ 0.05 was considered significant. P-value ˂ 0.001 was considered highly significant. P-value ˃ 0.05 was considered non-significant.

Table 5 and Fig 10 show the changes in oxidative and antioxidant enzymes in pancreatic tissues of control and treated male rats with MSCs, Q, or Q/Zn and their combinations. MDA levels of diabetic animals significantly increased with a decrease in antioxidant enzymes, namely SOD, CAT, GRx, and GST. A combination of MSCs and Q/Zn decreased the MDA levels and increased antioxidant levels more than treatment with MSCs or Q individually.

**Table 5:** Changes in oxidative/antioxidant parameters of antioxidant enzymes in pancreatic tissues of control and treated male rats with quercetin, quercetin/Zn, or stem cells and their combinations.

Groups	Parameters				
	Pancreatic Catalase (U/g)	Pancreatic SOD (U/g)	Pancreatic MDA (U/g)	Pancreatic GRx (U/g)	Pancreatic GST (U/g)
Control group	1.78 ± 0.21^a^	22.15 ± 1.15^ab^	3.45±0.48^g^	30.05 ± 1.85^a^	21.45 ± 0.82^a^
STZ group	0.46 ± 0.10^e^	7.22 ± 1.35^g^	77.15 ± 0.96^a^	9.56 ± 1.18^g^	5.45 ± 0.58^g^
STZ plus stem cell group	1.22 ± 0.36^d^	13.91 ± 1.58^f^	25.42 ± 1.02^b^	20.15 ± 1.15^f^	8.77 ± 0.18^f^
STZ plus Quercetin (Q) group	1.40 ± 0.65^c^	16.64 ± 2.28^e^	20.40 ± 1.47^c^	23.15 ± 1.15^e^	11.52 ± 1.55^de^
STZ plus Quercetin/Zn (Q/Zn) group	1.60 ± 0.53^b^	18.15 ± 1.35^d^	18.36 ± 1.26^d^	25.49 ± 1.91^d^	14.42 ± 0.47^d^
STZ plus Quercetin (Q) and stem cells	1.65 ± 0.48^b^	19.55 ± 2.16^c^	15.26 ± 1.45^e^	26.51 ± 1.28^c^	16.67 ± 0.66^c^
STZ plus Quercetin /Zn (Q/Zn) and stem cells	1.75 ± 0.22^a^	21.18 ± 2.25^b^	10.78 ± 1.25^f^	27.78 ±1.58^bc^	18.50 ± 0.58^b^

Means within the same column (mean ± SE) (n = 10) carrying different letters are significant at P ≤ 0.05. The highest value has the symbol (a) and decreasing in value were assigned alphabetically (While the same symbols are non-significant to each other’s and the different symbols are significant).

MPO and XO levels of rats treated with MSCs alone or in combination with Q and Q/Zn significantly decreased four weeks after MSC transplantation when compared with diabetic non-treated rats. Moreover, the decrease was significant compared with the Q/Zn treated group (Table 6).

**Table 6:** Changes in MPO and XO in pancreatic tissues of control and treated male rats with quercetin, quercetin/Zn, or stem cells and their combinations.

Groups	Parameters	
	MPO (nmol/min/mL)	XO (U/g)
Control group	16.16 ± 1.36^d^	17.25 ± 1.45^g^
STZ group	26.16 ± 1.35^a^	33.55 ± 1.36^a^
STZ plus stem cell group	20.24 ± 1.45^b^	26.15 ± 1.25^b^
STZ plus Quercetin (Q) group	18.19 ± 1.36^c^	24.35 ± 1.25^c^
STZ plus Quercetin/Zn (Q/Zn) group	21.56 ± 2.06^b^	22.17 ± 3.52^d^
STZ plus Quercetin (Q) and stem cells	16.55 ± 1.29^d^	20.65 ± 1.36^e^
STZ plus Quercetin /Zn (Q/Zn) and stem cells	15.48 ± 2.16^e^	18.16 ± 1.39^fg^

Means within the same column (mean ± SE) (n = 10) carrying different letters are significant at P ≤ 0.05.

The highest value has the symbol (a) and decreasing in value were assigned alphabetically.

The MPO and XO levels of the treated-rats with MSCs alone or in combination with Q and/or Q/Zn revealed a significant decrease in four weeks after MSCs transplantation when compared with diabetic non treated rats. Also, they showed significantly diminished as compared to the treated-group with Q/Zn (Table 6).

### Histological examination

**Fig 11:**
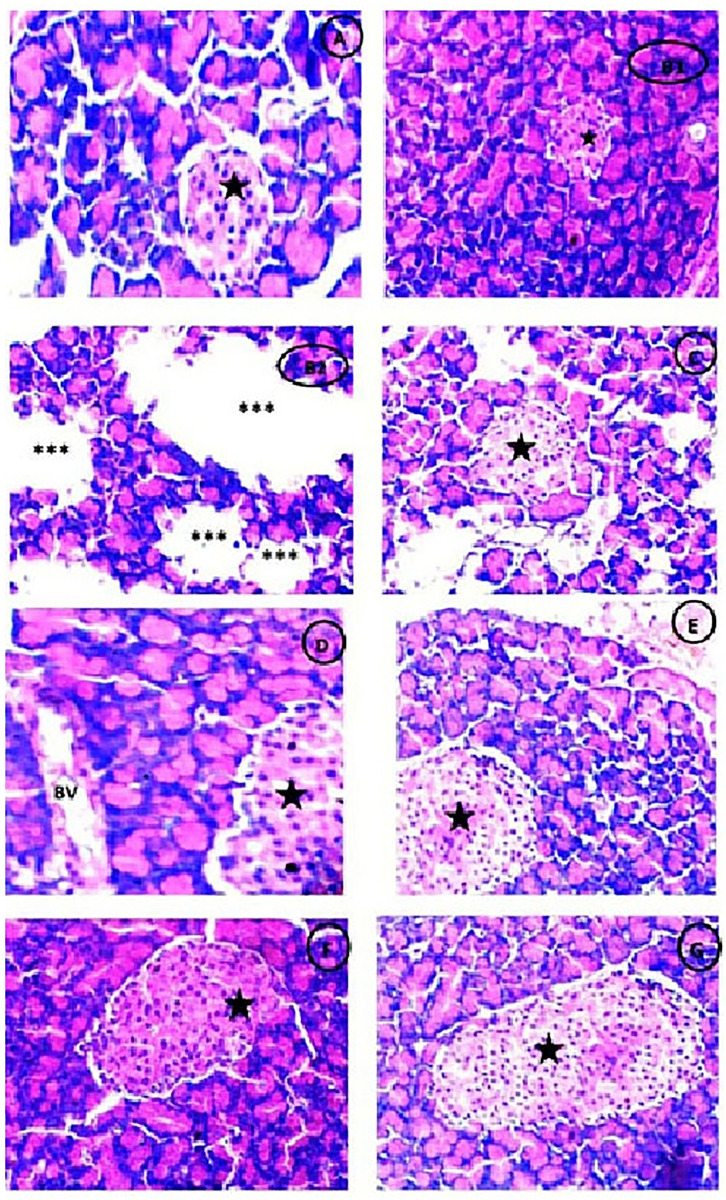
Photomicrograph of Pancreas showing (A) Normal pancreatic parenchyma and normal appearance of Islets of Langerhans (Black star) (H &E X400). (B1,2) STZ treated group showing large detached pancreatic parenchyma (***) with some parts showing very reduced islets of Langerhans (Black star) (H &E X400).(C) STZ plus stem cell group showing partial restoration of detached pancreatic parenchyma with mild sized islets of Langerhans (Black star) (H &E X400). (D) STZ plus (Q) group showing normal pancreatic parenchyma with appearance of blood vessels (BV) and mild enlarged islets of Langerhans (Black star) (H &E X400).(E) STZ plus (Q/Zn) group showing intact pancreatic parenchyma with mild enlarged islet of Langerhans (Black star) (H &E X400).(F) STZ plus (Q) and stem cells showing highly intact pancreatic parenchyma with enlarged size of islet of Langerhans (Black star) than group treated with Q only (H &E X400). (G) STZ plus (Q/Zn) and stem cells showing more high intact pancreatic parenchyma with enlarged size of islet of langerhans (Black star) more noticed than other treated groups (H &E X400).

Photomicrograph of Pancreatic tissues showing normal pancreatic parenchyma and normal appearance of Islets of Langerhans in control group (Fig 11A), while appearance of large detached pancreatic parenchyma with some reduced islets of Langerhans in diabetic (STZ) untreated group (Fig 11B1 and 11B2). Meanwhile, there was partial restoration of detached pancreatic parenchyma with mild sized islets of Langerhans with intact pancreatic parenchyma in both diabetic groups treated with either (Q) or (Q/Zn) (Fig 11C and 11D). There was great improvement in diabetic groups treated with stem cells (Fig 11E) and/or Q or Q/Zn (Fig 11F and 11G), but in the group of treatment with combination of MSCs and Q/Zn, there was highly intact pancreatic parenchyma with enlarged intact fit size of islets of Langerhans more noticed (H & E, X400). The histological index scoring for clarifying the pancreatic structural changes as shown in Table 7.

**Table 7:** Histopathological findings in pancreatic tissues of normal rats, diabetic non treated rats and diabetic treated rats with either quercetin, quercetin/Zn, or stem cells separately or in combination.

Findings	Control group	STZ group	STZ plus stem cell group	STZ plus (Q) group	STZ plus (Q/Zn) group	STZ plus (Q) plus stem cells	STZ plus (Q/Zn) plus stem cells
Normal pancreatic parenchyma	++++	------	- - ++	++++	++++	++++	++++
Detached pancreatic parenchyma	------	++++	- - ++	------	------	------	------
Normal sized islets of Langerhans	++++	------	- - ++	++++	- - ++	-+++	++++
Reduced islets of Langerhans	------	++++	------	------	------	------	------
Mild sized islets of Langerhans	------	- - - +	-+++	-+++	- - ++	- - - +	- - - +
Enlarged size of islet of Langerhans	------	------	- - - +	- - - +	- - ++	-+++	++++

------Absence of the change in the animals of the studied group.

++++ A change was observed in 90% the group.

-+++ A change was observed in 80% the group.

- - ++ A change was observed in 50% of the group.

- - - + A change was observed in 25% of the group.

**Fig 12:**
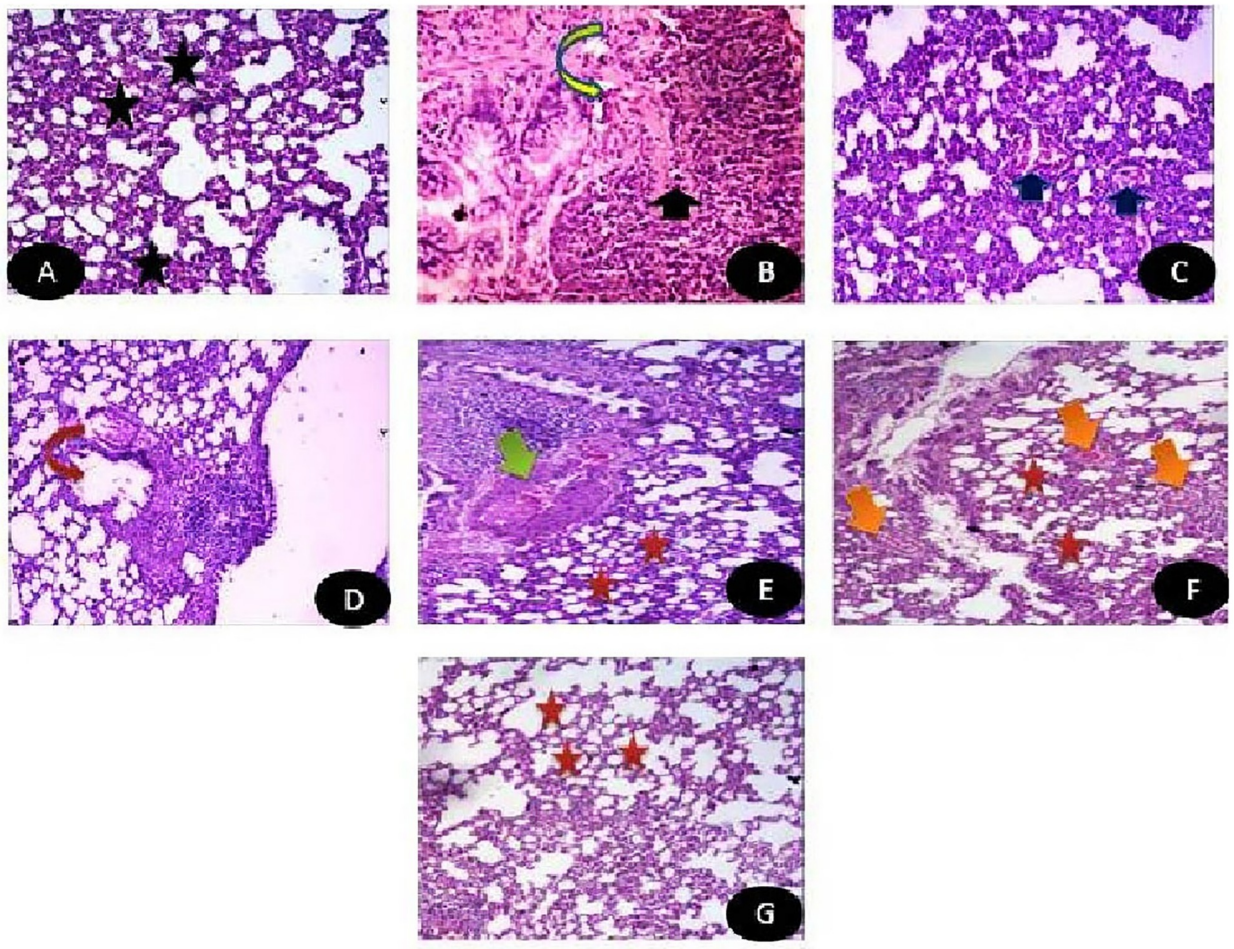
Photomicrograph of Lung tissues showing (A) Control group: Normal lung parenchyma (H&E X400). (B) STZ group: Lung tissues showing dense aggregates of chronic inflammatory cells (Black arrow) with appearance of some necrotic areas (inverted yellow arrow) (H&E X400). (C) STZ+ stem cells: Lung tissues with mild improvement with appearance of mild interstitial inflammation (Blue arrow) (H&E X400). (D) STZ+Q: Lung tissues showing normal alveolar walls with mild emphysematous changes (inverted red arrow) (H&E X400). (E) STZ + Q/Zn showing pulmonary parenchyma within normal structure (Red star) with little reactive hyperplasia of submucosal lymphoid tissues (Green arrow) (H&E X400). (F) STZ+ Q+ stem cells showing normal pulmonary parenchyma with in some hyperplasia of some lymphoid tissues (Orange arrow) (H&E X400).(G) STZ+Q/Zn+ stem cells showing pulmonary parenchyma within almost normal structure with very mild enlargement in air spaces (Red star) (H&E X400).

Photomicrograph of Lung tissues showing Normal lung parenchyma in control group (Fig 12A). While there was appearance of dense aggregates of chronic inflammatory cells with presence of some necrotic areas in diabetic (STZ) untreated group (Fig 12B). Meanwhile, there was a great improvement in pulmonary structures in diabetic groups treated with either MSCs or Q/Zn. Diabetic groups treated with either Q (Fig 12C) showing mild improvement in pulmonary tissues with appearance of normal alveolar walls with mild emphysematous changes (which mean a chronic, irreversible disease of the lungs characterized by abnormal enlargement of air spaces in the lungs accompanied by destruction of some tissue lining the walls of the air spaces and this may be due to STZ action) (Fig 12D). While, diabetic group treated with Q/Zn showing pulmonary parenchyma within normal structure with little reactive hyperplasia of submucosal lymphoid tissues (Lymphoid hyperplasia is an increase in the number of normal cells (called lymphocytes) that are contained in lymph nodes (Fig 12E). This almost often happens when there is an infection with bacteria, viruses, or any sort of inflammation and is part of the body’s reaction to the infection). Eventually, Diabetic groups treated with MSCs and Q showing normal pulmonary parenchyma with in some hyperplasia of some lymphoid tissues (Fig 12F), while combination between MSCs and Q/Zn showing pulmonary parenchyma within almost normal structure (Fig 12G) (H & E, X400).

The histological index scoring for clarifying the pulmonary structural changes as shown in Table 8.

**Table 8:** Histopathological findings in pulmonary tissues of normal rats, diabetic non treated rats and diabetic treated rats with either quercetin, quercetin/Zn, or stem cells separately or in combination.

Findings	Control group	STZ group	STZ plus stem cell group	STZ plus (Q) group	STZ plus (Q/Zn) group	STZ plus (Q) plus stem cells	STZ plus (Q/Zn) plus stem cells
Normal lung parenchyma	++++	------	- - - +	- - ++	- - ++	-+++	++++
Chronic inflammatory cells	------	-+++	- - - +	- - - +	- - - +	- - - +	- - - +
Necrotic areas	------	++++	- - - +	- - - +	- - - +	------	------
Improvement in lung tissues	++++	------	- - ++	-+++	-+++	++++	++++
Normal alveolar walls	++++	------	- - ++	++++	++++	++++	++++
Emphysematous changes	------	++++	++++	- - - +	- - - +	------	------
Lymphoid hyperplasia	------	++++	- - - +	- - - +	- - - +	- - - +	------

------ Absence of the change in the animals of the studied group.

++++ A change was observed in 90% the group.

-+++ A change was observed in 80% the group.

- - ++ A change was observed in 50% of the group.

- - - + A change was observed in 25% of the group.

### Illustrative 3D parametric ECG

**Fig 13:**
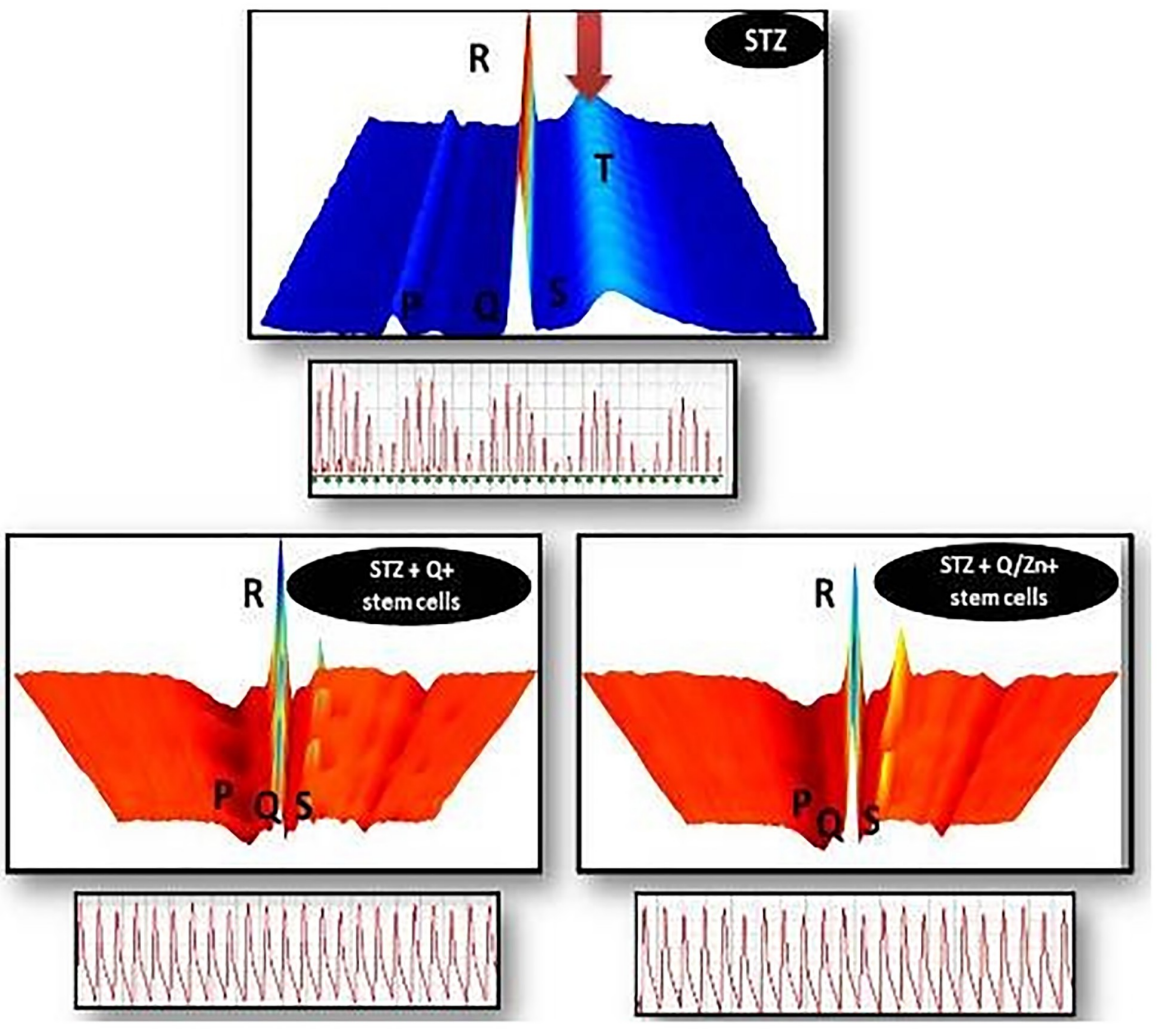
Illustrative 3D parametric ECG displays radial velocity with the lab chart software. STZ group showing blue color, which indicates incidence of oxidative stress, and highly wavy (P and T) waves, with the appearance of faint blue color, which indicates cardiac cell necrosis and area of non-clearance flow with more irregular electrocardiogram. STZ + Q + stem cells showing normal heart beat, with characteristic points (P,Q,R, S) and normal signal as (P) represents contraction of the heart and movement of blood from up down the heart, (QRS) represents ventricular contraction and appears above other signals, (T) represents the relaxation of the heart and pulse end with normal waves and appearance of rich oxygenated red color with less oxidative stress damages with mild waves. STZ + Q + stem cells showing normal heart beat with characteristic points (P,Q,R, S) and normal waves and appearance of rich oxygenated red color with less oxidative stress damages with more regular waves than Q alone with stem cell with normal electrocardiogram.

Illustrative 3D parametric ECG displays of radial velocity using the LabChart software, The color scale, used to accentuate the “surface geography” of the 3D plot, goes from cooler (blues) to warmer (reds) colors over the range from low to high oxygenated blood: the STZ group exhibiting oxidative stress and appearance of cardiac cell necrosis with appearance of color scale of dark blue, that is mean low oxygenated blood, The surface plot of the T-waves of this sample shows the T-waves “wavy” not in sequence manner, Beats also are “wavy” in sequence, that is mean that the beats aren’t regular. STZ + Q + stem cells exhibiting normal heart beat with characteristic points (P,Q,R, S), and STZ + Q/Zn + stem cells showing normal heart beat with characteristic points (P,Q,R,S), normal waves and appearance of warmer red colour that’s mean rich oxygenated red blood, with less oxidative stress damage, The surface plot of the T-waves of this sample shows the T-waves “stacked” in sequence one behind the other, Beats also are “stacked” in sequence one behind the other as shown in (Fig 13).

### TEM examination and great restoration of pancreatic structure by combination between stem cells and Q/Zn complex

**Fig 14:**
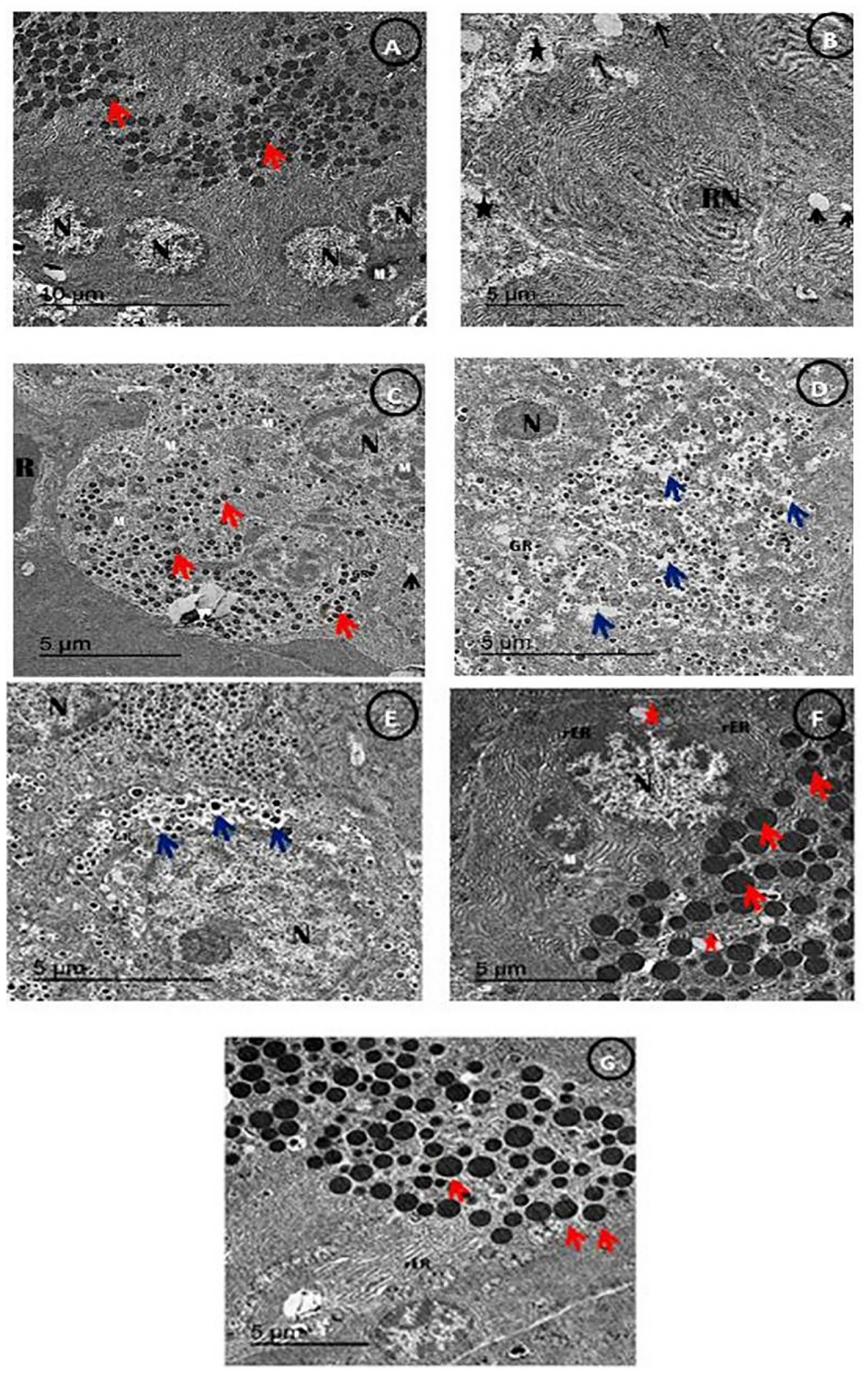
An electron micrograph of β-cells of the pancreatic tissues showing (A) control group showing β-cells with normal distinct intracellular spaces showing normal multi euchromatic rounded nuclei (N), normal sized mitochondria (M) with appearing of normal sized β-granules with an electron dense core (Red arrow) (Scale bar = 10μm). (B) STZ treated group showing detached pancreatic parenchyma (Black star) with reduced nucleus (RN) with disappearance of β-granules and appearance of some empty granules (Black arrow) with reduced sized mitochondria (Black arrow) (Scale bar = 5 μm). (C) STZ plus stem cell group showing restoration of pancreatic parenchyma with appearance of some small sized β-granules (Red arrow) with appearance of β-granular area (GR) with remaining of some Red blood corpuscles (R) and enlarged size mitochondria (M) than STZ group with appearance of nucleus (N) and appearance of some vacuoles (V) (Scale bar = 5 μm).(D) STZ plus (Q) group showing restoration of pancreatic parenchyma with appearance of multi small sized β-granules (Red arrow) with appearance of increased β-granular area (GR) with appearance of some vacuoles (Blue arrow) with appearance of euchromatic nucleus (N) (Scale bar = 5 μm). (E) STZ plus (Q/Zn) group showing restoration of pancreatic parenchyma with appearance of multi small sized β-granules and some enlarged sized β granules (Blue arrow) with appearance of increased β-granular area (GR) with appearance of some vacuoles (Blue arrow) with appearance of enlarges euchromatic nucleus (N) (Scale bar = 5 μm). (F) STZ plus (Q) and stem cells showing condensed euochromatic nucleus (N), with appearance of more rough endoplasmic reticulum (rER) and multi enlarged β-granules of islet of Langerhans (Red arrow) with normal sized mitochondria (M) and decreasing of β-granular spaces (Scale bar = 5 μm).(G) STZ plus (Q/Zn) and stem cells showing more restoration of β-granules of islet of langerhans with more condensed and enlarged β-granules and decreasing granular area space and appearance of rough endoplasmic reticulum (Scale bar = 5 μm).

Fig 14, An electron micrograph of β-cells of Pancreas showing normal β-cells showing normal multi euchromatic rounded nuclei, normal sized mitochondria with appearing of normal sized β-granules with an electron dense core in the control group (Fig 14A). Meanwhile, There was detached pancreatic parenchyma with reduced nucleus with disappearance of β-granules and appearance of some empty granules with reduced sized mitochondria in the diabetic untreated group (Fig 14B). While, diabetic group treated with MSCs showing restoration of pancreatic parenchyma with appearance of some small sized β-granules (Fig 14C). While, Diabetic group treated with either Q and/or Q/Zn showing restoration of pancreatic parenchyma with appearance of multi small sized β-granules with appearance of increased β-granular area with appearance of some vacuoles with appearance of euchromatic nucleus and the great restoration was noticed in Q/Zn treated group (Fig 14D and 14E). Diabetic group treated with MSCs in combination with Q and/or Q/Zn showing restoration of the condensed euochromatic nucleus, with appearance of more rough endoplasmic reticulum with normal sized mitochondria and decreasing of β-granular spaces (Fig 14F) and the more restoration was recorded in Q/Zn treated group in combination with MSCs with appearance of enlarged β-granules and appearance of rough endoplasmic reticulum (Fig 14G) (Scale bar = 5μm).

### Comet assay and great reduction in genotoxicity induced by STZ by combination between stem cells and Q/Zn complex

**Fig 15:**
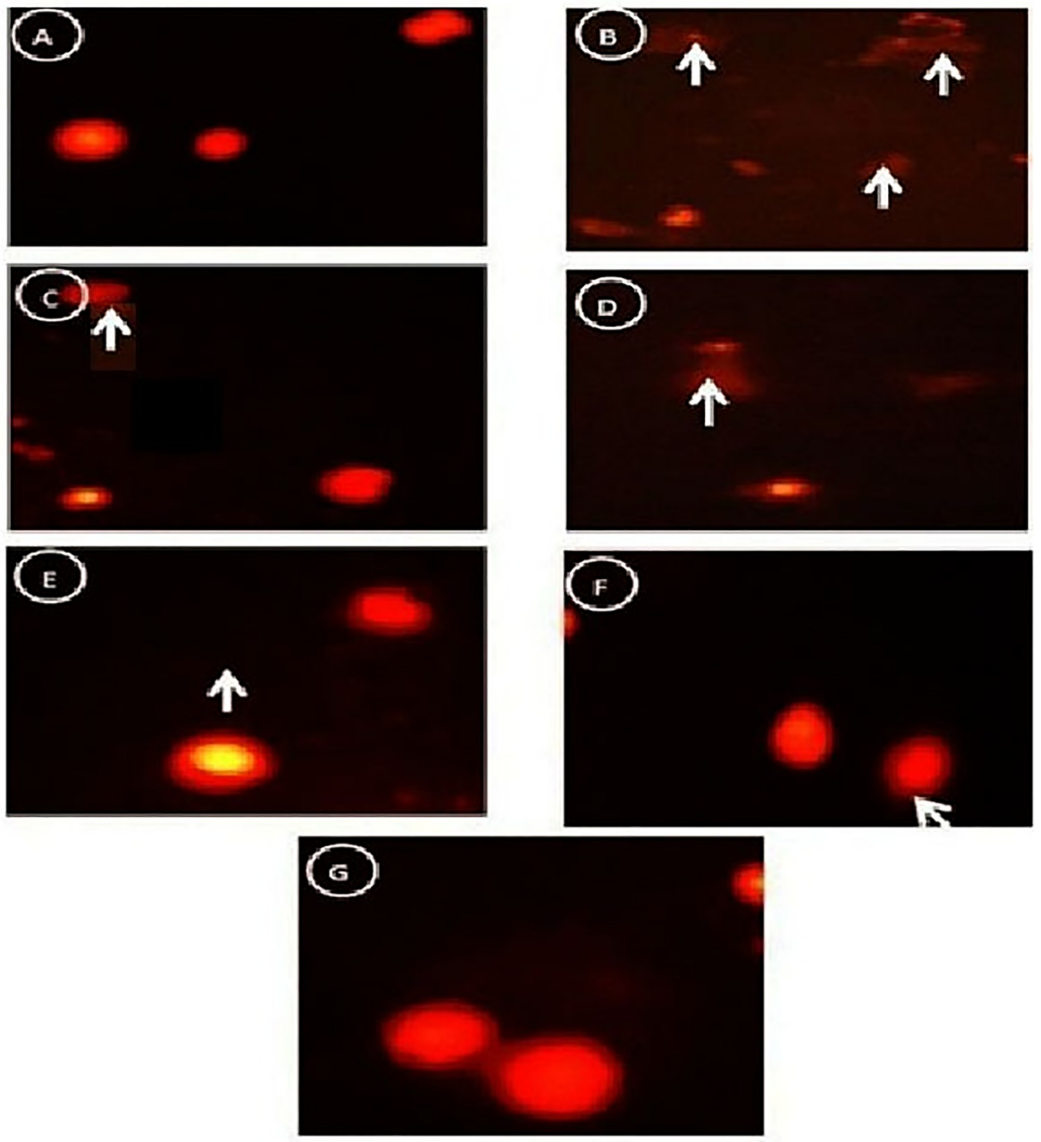
(A) Control group showed normal round cells with intact nuclei. (B) STZ group showed greater damage, as evidenced by the presence of more than two apoptotic cells with the appearance of a large hollow area near the tail and a small head (comet). (C) STZ + stem cells group showed some intact nuclei and some apoptotic cells. (D) STZ + Q group showed lower DNA damage, as evidenced by fewer damaged nuclei and with a hollow area (tail). (E) STZ + Q/Zn group showed lower DNA damage and fewer damaged nuclei. (F) STZ + Q and stem cells group showed higher amelioration and lower DNA damage. (G) STZ + Q/Zn and stem cells group showed more intact cells and fewer comet cells and undamaged DNA.

Fig 15 Comet images of the pancreatic tissues was performed to clarify the genotoxicity by appearance of round cell with tail hollow or appearance of non- genotoxicity by appearance of normal round cell without tail hallow (Fig 15A) control group showed intact nuclei and normal round cell without tail hallow. (Fig 15B) STZ group showed high degree of damage with appearance of 2 apoptotic cells (White arrow) with large tail hallow and small head in the form a comet-shaped. (Fig 15C) STZ plus stem cell group showing some intact nuclei but with apoptotic cells (White star). (Fig 15D) STZ plus Q group clarified less damaged DNA strands which are confirmed by less damaged nuclei as mild cell contains a tail appear as a hallow area (White arrow).(Fig 15E) STZ plus Q/Zn group which showed less damaged DNA strands and less damaged nuclei (White arrow). (Fig 15F) STZ plus Q and stem cells which showed high amelioration in the cells as recorded less tail length and decreasing of the percent of damaged DNA (White arrow). (Fig 15G) STZ plus Q/Zn and stem cells clarified more % of the intact cells with less comet cells and undamaged DNA. Table 9 shows effect on oxidative DNA damage level (Tail length, DNA% and tail moment) and apoptotic cell population (apoptosis %) in control and treated male rats with Q, Q/Zn and/or stem cells and their combinations.

**Table 9:** The effect on oxidative DNA damage level (tail length, DNA% and tail moment) and apoptotic cell population (apoptosis %) in Pancreas of control and treated male rats with quercetin, quercetin/Zn, or stem cells and their combinations.

Group	Tail Length (px)	%DNA in Tail	Tail Moment (Units)	Apoptosis %
Control group	3.10±0.48^e^	2.15±0.26^g^	0.352±0.02^g^	8.714±1.01^g^
STZ group	9.906±1.27 ^a^	25.73±2.04 ^a^	7.5987±1.12 ^a^	89.148±3.45^a^
STZ plus stem cell group	5.140±1.72 ^b^	17.879±2.41 ^b^	3.7657±1.71 ^b^	59.14±2.15^b^
STZ plus Quercetin (Q) group	4.448±1.15 ^c^	11.714±1.47^c^	2.5784±0.64 ^c^	39.374±2.59^cd^
STZ plus Quercetin/Zn (Q/Zn) group	4.146±0.47 ^c^	8.124±0.87 ^d^	2.3480±0.07^de^	37.575±1.65^d^
STZ plus Quercetin (Q) and stem cells	4.006±0.68 ^c^	5.416±1.36 ^e^	2.1322±0.24 ^e^	17.583±3.25^e^
STZ plus Quercetin /Zn (Q/Zn) and stem cells	3.189±0.78 ^de^	3.407±1.16 ^f^	0.8572±0.24 ^f^	10.572±2.75^f^

Values are expressed as means ± SE; n = 10 for each treatment group.

### Gene expression levels in the lung of treated groups

**Fig 16:**
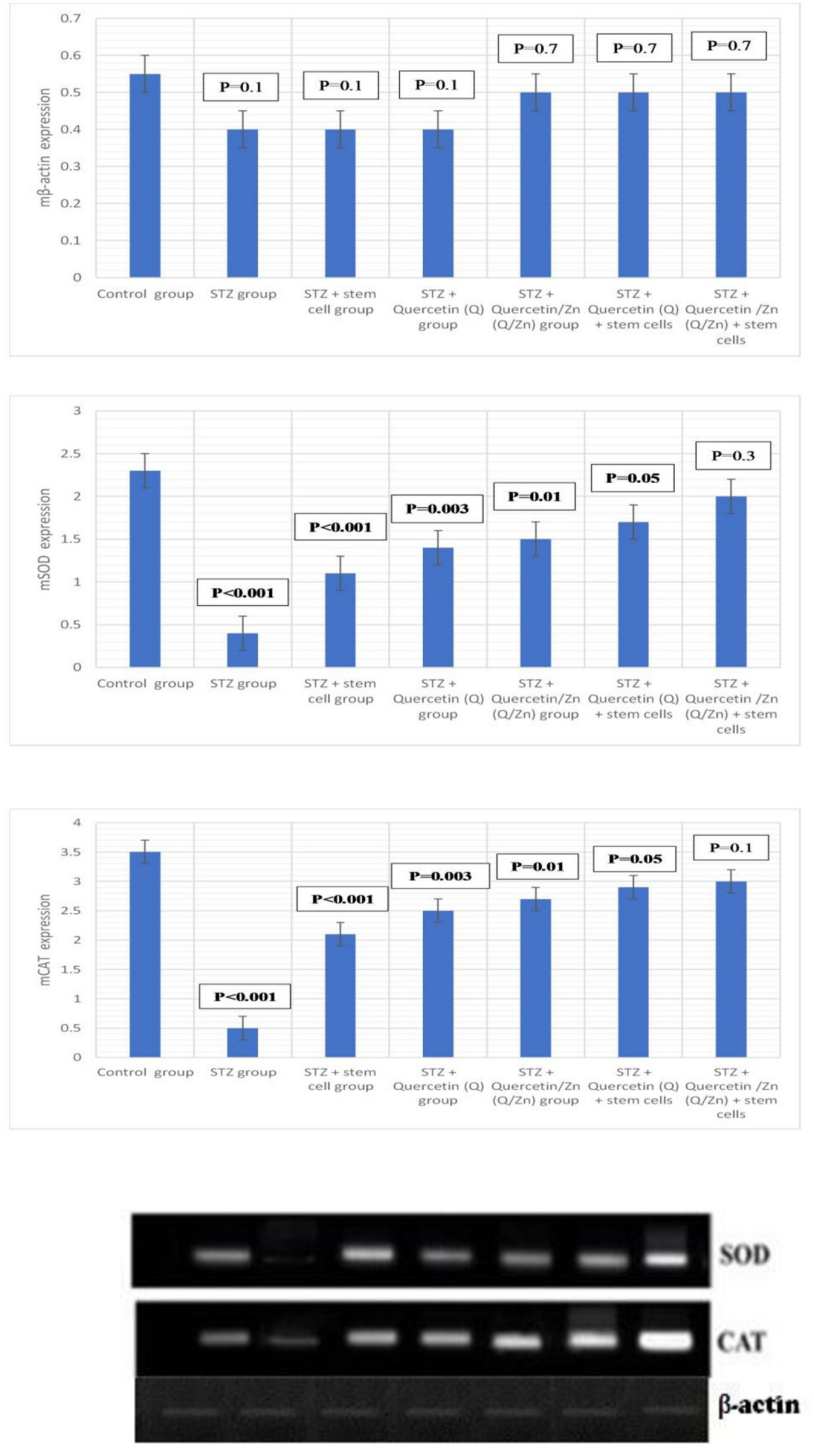
Gene expression of mCAT, mSOD and mβ-actin in lung tissues (mean ±S.E) (n = 8). Autoradiograms for RT-PCR products corresponding to the different groups in the experiment. Whereas, Represents the expression of SOD, CAT and β-actin. Where, P-value ≤ 0.05 was considered significant. P-value ˂ 0.001 was considered highly significant. P-value ˃ 0.05 was considered non-significant.

PCR analysis was performed to evaluate whether the inhibitory effects of MSCs, Q/Zn, or both on oxidative stress in pulmonary tissues was due to the regulation of antioxidant genes, such as SOD and CAT (Table 2). Relative mRNA expression of SOD and CAT was lower in diabetic untreated rats than controls. In the diabetic group treated with MSCs plus Q/Zn, relative levels of SOD and CAT mRNA significantly increased compared with that in the STZ group (Fig 16).

## Discussion

Diabetes mellitus is a major public health concern globally. Diabetes mellitus is a metabolic syndrome resulting from insulin deficiency, insulin resistance, or both [38]. According to the WHO, diabetes mellitus is a silent killer, affecting 1%–5% of the global population [39]. By 2025, the number of patients with diabetes is expected to exceed 325 million. Therefore, new therapies and drugs should be urgently developed [40]. Oxidative stress plays an important role in the development of diabetes mellitus and its associated complications [21].

We used MSCs, Q/Zn, or both to treat rats with diabetes induced by STZ. We evaluated the pros and cons of each treatment separately and in combination. The novelty of this study includes use of a combination of treatments against pancreatic complications in diabetes. The novel combination of Q/Zn and stem cell therapy may reduce COVID-19-mediated inflammatory response in pulmonary tissue, especially in patients with diabetes.

Diabetic non-treated animals had elevated blood glucose, HbA1c, and lipid profile (TG, TC, LDL-C, and vLDL-c), except HDL-c, which decreased. Insulin and C-peptide levels were markedly reduced. Moreover, levels of pancreatic MDA, MPO, and XO were elevated, and antioxidant enzymes (CAT, SOD, GRx, and GST) significantly decreased. Hyperglycemia causes severe oxidative stress damage that accelerates the development of diabetes mellitus. Relative expression of CAT and SOD mRNA decreased in STZ-treated rats compared to control and other treated groups. TEM, histopathological evaluation, and DNA-damage analysis of pancreatic tissues revealed a decrease in the number and size of islets of Langerhans, and comet-shaped apoptotic cells, with a large hollow tail and small head, were observed with detached pancreatic parenchyma. Pulmonary tissues exhibited severe damage; aggregates of chronic inflammatory cells and some necrotic areas in STZ (diabetic untreated group) were observed compared to the control and other treated groups. Cell and DNA morphology greatly improved in diabetic groups treated with either stem cells, Q/Zn, or both. Illustrative 3D parametric ECG revealed severe oxidative stress in cardiac tissue. meanwhile, appearance of normal heart beats with normal ECG in diabetic groups treated with either stem cells, Q/Zn, or both.

STZ destroys β-cells of islets of Langerhans in pancreatic tissue, resulting in elevation of blood glucose level, and thus, inhibition of insulin secretion. These effects are mostly due to reduced glucose entry into adipose tissue and muscle as well as increased glycogen breakdown and glucose production by the liver [41]. It also exhibited degenerative morphological alterations and reducing of the pancreatic tissue islets through mechanisms, including the production of reactive oxygen species and induction of inflammation. These findings coincide with the results of other studies [42].

In this study, treatment with MSCs or Q/Zn alone or in combination significantly improved all parameters compared with that in the diabetic non-treated group. Moreover, combined treatment was better than each alone.

Lipid profile (TG, TC, LDL-c, and VLDL-c) levels decreased markedly after treatment with MSCs and Q/Zn as compared to that in the diabetic non-treated group. This decrease could be because of the effect of MSCs, which could be due to increasing insulin that activated the lipoprotein lipase and due to the potential anti-diabetogenic and hypocholesterolemic effect of Q/Zn. However, Q/Zn lowered TG, TC, LDL-c, and VLDL-c levels in the diabetic treated group. The combination of MSCs and Q/Zn for treating hyperglycemic rats greatly decreased TG, TC, LDL-c, and VLDL-c levels compared to other treatments. Thus, Q/Zn improves the performance of MSCs in decreasing cholesterol levels.

Decreasing adipogenesis and elevating LDL receptor expression could decrease TG, which increases lipoprotein lipase activity [43]. Our results confirmed the ameliorative effect of the Q/Zn complex in combination with MSCs on serum lipids levels. In this study, the lipid profile pattern was optimized for treating diabetic rats with MSCs. Our results are consistent with those of Ahmed et al. [44], who reported that MSCs improved lipid profile by improving β-cell function and decreasing insulin resistance.

Treatment with Q or Q/Zn either alone or in combination with stem cells decreased TG and TC levels, thus improving the symptoms of diabetes by maintaining the lipid profile. Additionally, oral Q treatment for 8 weeks significantly reduced TC levels [45], consistent with our results, which revealed the reduction in both TG and TC after treatment with Q, Q/Zn, or both.

In this study, treatment with both MSCs and Q/Zn significantly reduced blood glucose level. The decrease in HbA1c was statistically significant. Furthermore, these changes were concurrent with the increase in insulin level. Our results are consistent with those from other studies, which have suggested potential mechanisms to explain the role of MSCs in controlling blood glucose. MSCs can differentiate into islet-like insulin-producing cells (IPCs), promote the restoration of β-cells in the pancreatic islet and protect endogenous β-cells against oxidative stress through immunotherapeutic mechanisms, thereby secreting more insulin, which aids in lowering blood glucose. These data are consistent with those from other studies [46, 47]. MSCs help regenerate endogenous β-cells in pancreatic islets by secreting specific cytokines and growth factors. Si et al. [6] reported that in diabetic rats, MSC infusion resulted in significant regeneration of endogenous β-cells. Moreover, MSC infusion significantly increased insulin sensitivity as evidenced by phosphorylated insulin receptor substrate 1, protein kinase B, and insulin target tissue GLUT4 [48]. Lee et al. [49] reported an improvement in regenerated mouse pancreatic islet β-cells following the transfer of BM-MSCs into diabetic mice.

Flavonoids have strong antidiabetic effects. The flavonoid Q exhibits strong hypoglycemic effects and insulin-sensitizing activity by promoting pancreatic β-cell regeneration and insulin secretion [50]. These results are consistent with ours—lower blood glucose levels accompanied with higher insulin and serum C-peptide levels in diabetic groups treated with Q, Q/Zn, or both either alone or in combination with stem cells. Studies have reported the antidiabetic effects of Q and corroborate the capacity of the novel Q/Zn complex. Youl et al. [51] reported that Q administration promoted glibenclamide-induced insulin secretion and protected β-cells from oxidative damages [51].

The protective effects of Q on islet cells can be divided into three categories: increase in insulin secretion, protection of β-cells, and enhancement of β-cell regeneration. Our results are consistent with these categories, proving the efficiency of the proposed novel complex [52].

Q exhibits many pharmacological properties, including antidiabetic effects. Therefore, this motivated us to investigate the antidiabetic potential of the novel Q/Zn complex. Q/Zn exhibits many pharmacological effects in treating diabetes mellitus. The potential of the Q/Zn novel complex in preventing β-cell dysfunction associated with diabetes mellitus must be further investigated.

Q neutralizes free radicals in the body and protects cellular membranes from damage [53]. Moreover, Q has antioxidant, anti-inflammatory, anti-cancer, and anti-infection activity [53]. Zn is the most vital part of our novel, which reinforces the antidiabetic and antioxidant capacities of Q. Zn is a vital element involved in the formation of β-cells of islet of Langerhans, and Zn deficiency correlates with the development of diabetes mellitus and glycemic complications [54]. Studies have analyzed the roles of Zn homeostasis in other endocrine diseases, with focus on Zn transporter function [55].

Stem cell therapy has many limitations. Studies have reported that stem cell injections suppress the requirement of exogenous insulin and antidiabetic drugs. However, we discovered that Q/Zn was effective in combination with stem cell therapy, although Q/Zn alone produced satisfactory results. MSC and Q/Zn therapy may be the best option for patients with diabetes with severe comorbidities, such as pneumonia, to first control the glycemic state and then regenerate lung tissues to block the inflammatory response. Thus, alleviating severe inflammation and cytokine storm in patients with diabetes infected with COVID-19. The Q/Zn complex, in combination with MSCs, can improve pulmonary function in patients with diabetes.

The isolated MSCs used in this study revealed high expression of CD105, which was reported to be associated with hematopoiesis and cell migration and has a potential role in homing. We detected MSCs by fluorescent staining and found the isolated MSCs homing into pancreatic tissues. Studies have reported that the expression of classical markers, such as CD34, CD105, and CD29, differ between the stem and progenitor cells, which are endothelial cells or MSCs, which is consistent with our results, due to weak expression of CD34 and high expression of CD29. Different levels of expression of certain stem cell markers will help understand the differentiation potential of MSCs and which population can be utilized for regeneration of β-cells in pancreatic tissues [56].

Consequently, restoration of endogenous insulin secretion represents a vital step in preventing hyperglycemia and hypoglycemia and to minimize the pathological complications of diabetes and self-management of glycemia; however, protection against inflammatory infections is most important [49, 57]. These finding indicate that Q, Q/Zn, or both accompanied with MSCs could ameliorate HbA1c levels in patients with diabetes.

Zn deficiency is reported to impair glucose metabolism and disturb metabolic adaptation to meal-feeding in rats, thereby increasing insulin resistance, which may correlated with differences in the effect of insulin on glucose uptake and metabolism in these tissues. Fat cells require insulin for glucose uptake; therefore, if this process is blocked due to Zn deficiency, tissues would not adapt to meal-feeding and the incorporation of glucose into fatty acids would decrease [58]. This explains the success of the novel complex Q/Zn in alleviating insulin resistance, thereby improving the glycometabolic state and biochemical parameters related to adjusting the levels of blood glucose, insulin hormone, and C-peptide and lipid parameters, namely TC, TG, LDL-c, HDL-c, and v-LDL-c.

Olechnowicz et al. [59] reported that Zn plays a vital role in the development of metabolic syndrome and participates in the regulation of cytokine expression and may have a role in suppressing inflammation. Zn is required to reinforce antioxidant enzymes that scavenge reactive oxygen species, thereby reducing oxidative stress. Zn plays a key role in correction of lipid functioning and organization of glucose metabolism, thereby regulating insulin expression. Understanding the properties of Zn may help in treating metabolic syndrome, thus protecting against stroke and death. Thus, we propose the potential role of the novel Q/Zn complex in combination with MSCs in protecting patients with diabetes and cardiac complications, This strengthens the role of the novel Q/Zn complex in improving blood pressure, which was greatly appeared in 3D ECG that was clearly appeared by low oxidative stress and appearance of normal heart beats, normal level of blood glucose, and LDL cholesterol serum level.

Pancreatic β-cells contain higher Zn levels than other pancreatic cells. In particular, granules secreting insulin within β-cells have the highest Zn levels [11]. Our findings prove that Zn elevates the antidiabetic activity of Q and can be used to control hyperglycemia and activate β-cells in patients with diabetes. Moreover, Zn is essential for the synthesis and structural stability of insulin [60]. It is a hexamer of six insulin and two Zn molecules [61]; therefore, complex formation between Zn and Q increases the overall antidiabetic and antioxidant activities. This finding confirms our hypothesis regarding the safety of the novel complex in treating diabetes and its associated pathologies, including neuro and pulmonary complications.

Illustrative 3D parametric ECG displays of radial velocity with the LabChart software revealed normal heart beats with the characteristic points (P,Q,R, S) with normal waves and rich oxygenated red blood with less oxidative stress damage in the diabetic group treated with MSCs and Q/Zn. These finding are consistent with those of Sadraddin et al. [62], who revealed the role of MSCs as an antiarrhythmic agent and in maintaining blood pressure and normal heart beat in myocardial infarction. Moreover, they reported that treatment with MSCs resulted in shorter QRS duration and decreased the occurrence of ventricular tachycardia in the early period. The shortening of QRS indicates the ability of MSCs to improve cardiac electric conduction. This is consistent with our findings, which reveal normalizing QRS duration after MSC transplantation in combination with the novel complex Q/Zn.

The severity of oxidative stress can be determined in three ways: (i) by evaluation of ROS level, (ii) by direct evaluation of the concentration of antioxidants (enzymatic and nonenzymatic), and (iii) by measuring oxidative stress biomarkers, which are defined as biological molecules whose chemical existence is modified by ROS. Oxidative stress is the lack of antioxidant enzymes to quench ROS [63]. Studies have shown the ability of MSCs to reduce oxidative stress and increase the activity of antioxidant enzymes [64]; therefore, MSCs in combination with Q/Zn may increase the antioxidant capacity of patients with diabetes. We consider that the main way of MSC action relies on the activation of antioxidant enzymes and protection of extracellular pancreatic islet cells improved by the antioxidant and anti-inflammatory activity effects of MSCs.

Accordingly, our results revealed that the diabetes mellitus group (untreated diabetic control group) had elevated MDA level and decreased antioxidant enzyme levels (SOD, CAT, GRx, and GST). These results confirm the pivotal role of oxidative stress in diabetes mellitus. The proposed combination therapy with MSCs and Q/Zn elevated the antioxidant enzymes and improved pancreatic and pulmonary tissue structure. Similarly, permanent hyperglycemia may promote free radical production by auto-oxidation of lipid peroxides as well as dysfunction of the antioxidant defense system through the elevation of free radical levels, which induce oxidative stress [64].

Another important finding of this study was the significant increase in SOD and CAT mRNA expression in diabetic rats following treatment with MSCs and Q/Zn compared to that in diabetic untreated animals. Therefore, we hypothesized that both MSCs and Q/Zn improve diabetes-induced pancreatic complications, possibly by up regulating the expression of SOD and CAT mRNAs. This could be due to the anti-inflammatory and antioxidant properties of Q/Zn because studies have suggested an association between chronic inflammatory state and impaired insulin activity through the stimulation of insulin receptor expression on β-cells [65].

We confirmed gene expression of antioxidant enzymes in the lungs of diabetic untreated rats and diabetic rats treated with the novel combination. Madia et al. [66] assessed the levels of antioxidant enzymes. This change affected lung function and reduced lung dispensability against infection. These results illustrate the risk that patients with diabetes face when are infected with killer viruses, such as SARS-coV-2. Therefore, patients with diabetes require protective agents that improve innate immunity and enhance immune responses against life-threatening infections. The proposed combination therapy can increase the immunity of patients with diabetes against infections, as illustrated by 3D ECG, which shows less oxidative damage after Q/Zn administration. However, Sahebjami and Denholm [67] reported that lack of insulin in diabetes did not retard lung growth, although it reduced body weight. Because the effects of diabetes on both somatic and lung growth are associated with reduced body weight, we did not observe this change, as the lung was already affected regardless of body weight.

Histopathological and electron transmission results confirmed that diabetic groups treated with either MSCs, Q/Zn, or both noteworthy diminutive the degenerative alteration in the pancreatic islet β-cells and diminished the aggregates of the inflammatory cells and reduced necrotic areas. We have explained the ability of MSCs to differentiate into IPCs and renew pancreatic β-cells and assessed their role in modulating the immune system, in addition to the role of Zn and Q novel complex in improving the role of β-cells, reducing oxidative stress, and improving all biochemical parameters in both the pancreas and lungs.

In this study, the appearance of pancreatic inflammatory cells and reduction in the size of islets of Langerhans are the main signs of inflammation and damage, as reported [61]. These findings are consistent with that of Hassan et al. [68], who reported that pancreatic tissues exhibited marked damage and inflammation in the diabetic non treated group due to STZ toxicity and the complications of diabetes, whereas the pancreatic tissue of diabetic animals showed a marked improvement after treatment with MSCs. This improvement was in a strong harmony with the current histological examination finding in concurrent with the biochemical finding in which all of the parameters of organs functions were ameliorated in diabetic group treated with combination of MSCs and Q/Zn novel complex. In this study, the ameliorative effects of MSCs, Q/Zn, or both were confirmed by the improvement of pancreatic morphology, especially when both were used.

STZ enters β-cells through the glucose transporter and induces severe DNA-damage [69]. Our results corroborate this observation, the untreated diabetic group showed clear DNA-damage (genotoxicity), as evidenced by the appearance of comet cells (small head and hollow tail). However, combination therapy of MSCs and Q/Zn greatly alleviated STZ-mediated genotoxicity. Furthermore, the novelty of the combination therapy with MSCs and Q/Zn is that it can activate β-cells, which was confirmed using TEM and histological analyses of pancreatic tissue from the diabetic group treated with MSCs and Q/Zn, which presented larger β-cells and fewer comet cells. Therefore, the proposed combination therapy activated β-cells to increase insulin and C-peptide secretion.

Lung function is affected by diabetes. Schuyler et al. [70] reported that total lung function was reduced in juvenile diabetes due to the loss of lung elasticity, as exhibited by the appearance of pulmonary dysfunction in the diabetic nontreated group. Kida et al. [71] demonstrated significant morphological alterations in the lungs of diabetic rats, including thickening of alveolar walls and epithelia and enlargement of the basal lamina of alveolar capillaries. These findings confirm our histological results of the lungs of diabetic rats treated with the novel therapy, proving its effectiveness in treating diabetes-induced structural alterations of the lung.

*In vitro* studies have shown that Q inhibits lipopolysaccharides that induced interleukin (IL)-8 production in A549 cells, which are pulmonary cells, and this revealed the ability of Q in alleviating inflammation or cytokine storm, especially in pulmonary tissue. Moreover, Q inhibited the production of inflammatory enzymes (cyclooxygenase and lipoxygenase). Q exerted protective effects against H_2_O_2_-induced inflammation in some endothelial cells and downregulated CD80 expression in humans. Collectively, these finding confirmed the efficacy of the novel Q/Zn complex, especially after the addition of Zn to Q, which can act as a strong chelator of free radicals, thereby alleviating inflammation caused by infections, such as SARS-coV-2 [72].

Furthermore, Q possesses mast cell-stabilizing and gastrointestinal-cytoprotective activities, making it highly potent and safe. Q exerts a regulatory effect on immune cells, likely mediated through extracellular regulated kinase 2 and purified T lymphocytes [72]. Thus, the novel Q/Zn complex likely inhibits inflammation by downregulating or suppressing inflammatory pathways. In this context, the effects of the novel complex can be explained by the presence of Q, which acts as an anti-inflammatory agent by binding cellular receptors, as well as of Zn, which when combined with Q, acts as scavenger to combat viral infections like COVID-19 [72].

The respiratory viruses include rhino, corona, and influenza, which together have nearly 200 serotypes [73]. Similar symptoms can also be caused by mechanical irritation of the airways, allergy, or bacterial infections. Zn deficiency often accompanies increased susceptibility to infections [13]. This explanation supports and our data that the novel Q/Zn complex can combat inflammatory agents and protect pulmonary tissue, as seen through histological examination of pulmonary tissues in this study.

The challenge in diabetes is the urgent need to use safer compounds that have antidiabetic potential and activate and regenerate β-cells. Therefore, this study was designed to assess the protective effects of a novel combination therapy of MSCs and Q/Zn on oxidative damage, hyperglycemia, and inflammation as well as to evaluate the effectiveness of the novel therapy in treating pancreatic and pulmonary structural alterations to mimic the effects of diabetes mellitus. Therefore, the antioxidative, antigenotoxic, and anti-inflammatory capacities of Q/Zn, in combination with MSCs, could promote pancreatic islet cell survival, and thus, prevent or decrease the deterioration that results from diabetes mellitus or inflammatory inducers.

## Conclusion

Our findings revealed that pancreatic and pulmonary tissues were affected by the induction of experimental diabetes mellitus and clarified the potential of a novel combination therapy with Q/Zn and MSCs for diabetes mellitus, which improved insulin secretion, decreased cellular inflammation, and contributed to improving pancreatic and glycometabolic complications in diabetic rats than that when using MSCs or Q/Zn alone. Our results confirmed that the Q/Zn complex is highly efficient and safe for treating hyperglycemia, genotoxicity, and oxidative injury induced by diabetes mellitus. These results open new avenues to develop therapeutic strategies for diabetes mellitus and prevention of its associated complications, thereby providing hope for alleviating the symptoms of COVID-19 or other associated inflammatory disorders.

## Supporting information

S1 File (DOC)

S1 Raw images (PDF)

S2 Raw images (RAR)
