# Supplementary figures and images for: Quercetin/Zinc complex and stem cells: A new drug therapy to ameliorate glycometabolic control and pulmonary dysfunction in diabetes mellitus: Structural characterization and genetic studies
Source: PLoS One. 2021 Mar 4;16(3):e0246265. doi: 10.1371/journal.pone.0246265 (PMC7932096; doi:10.1371/journal.pone.0246265)

STZ (Sigma Aldrich) injection and induction of Diabetes mellitus


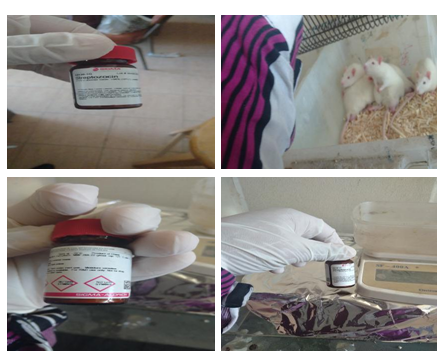


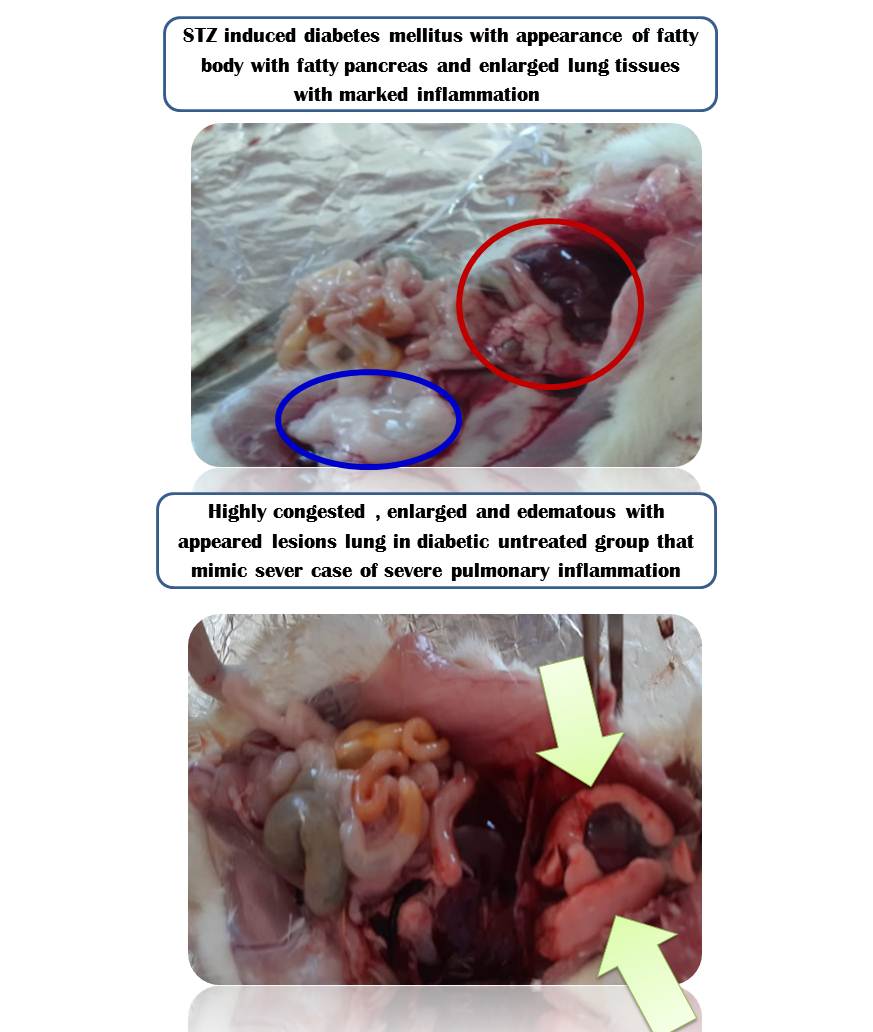


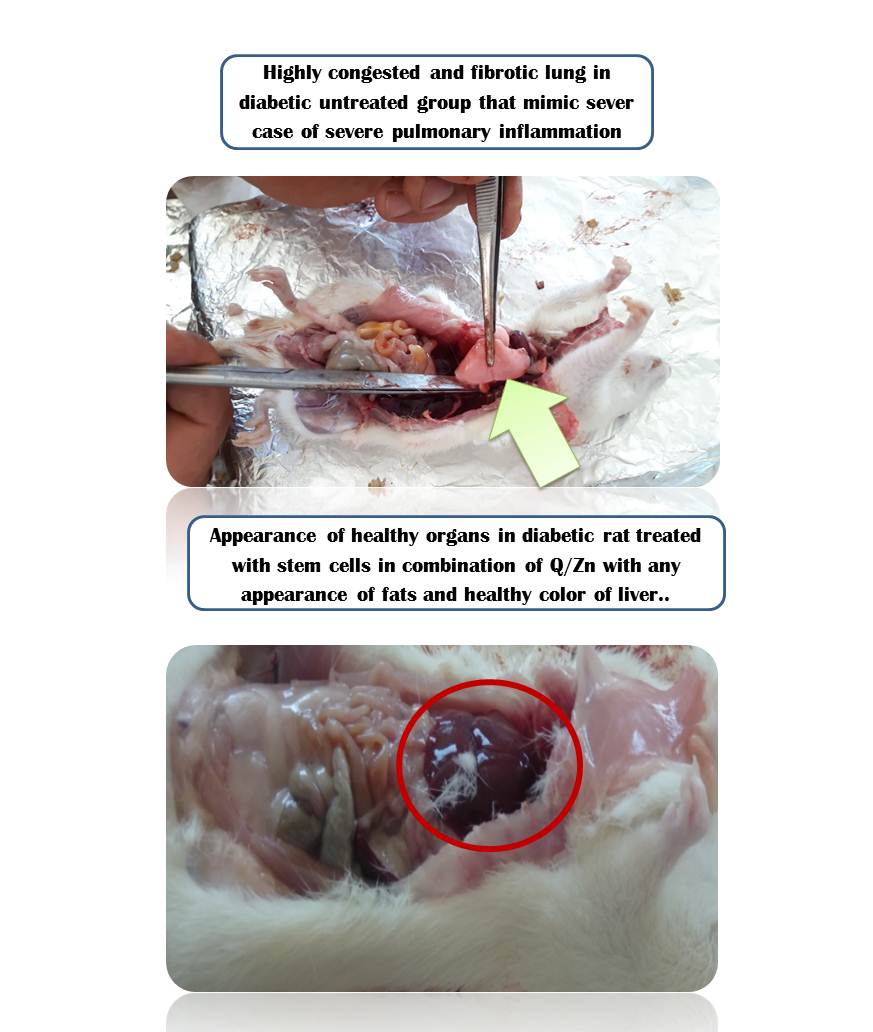


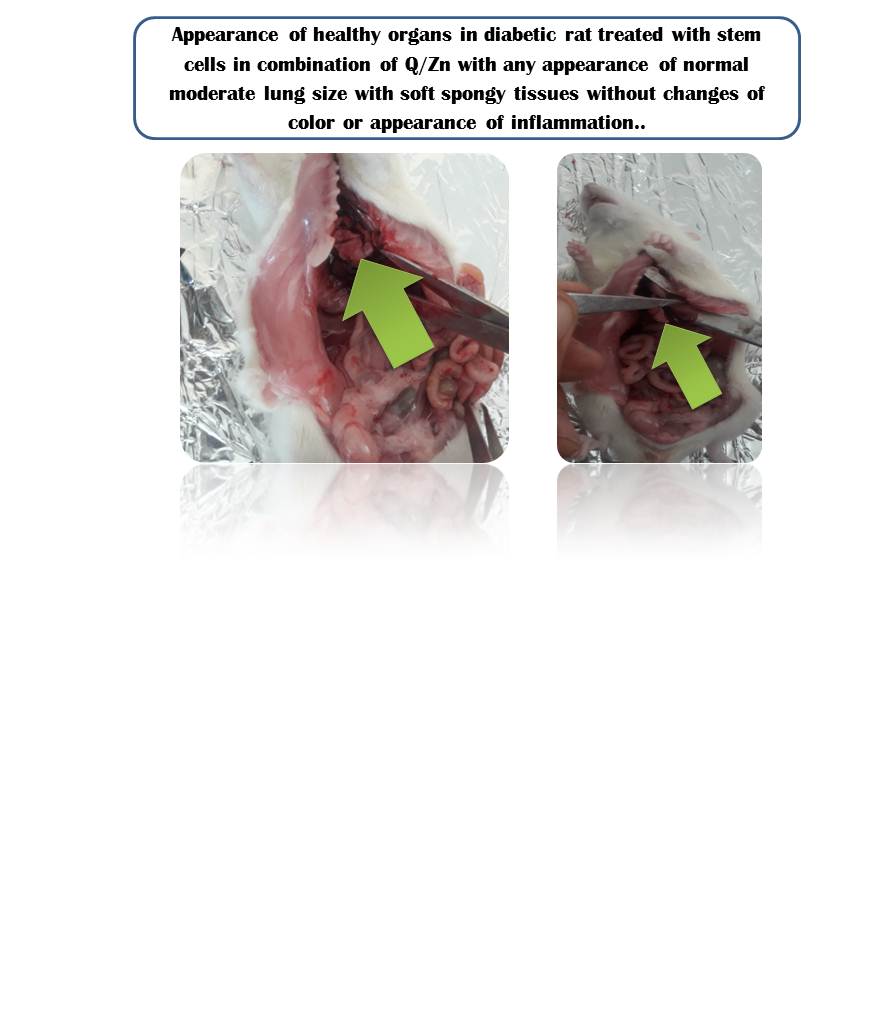

Supplement: S1 File — (DOC) [file pone.0246265.s001.doc]

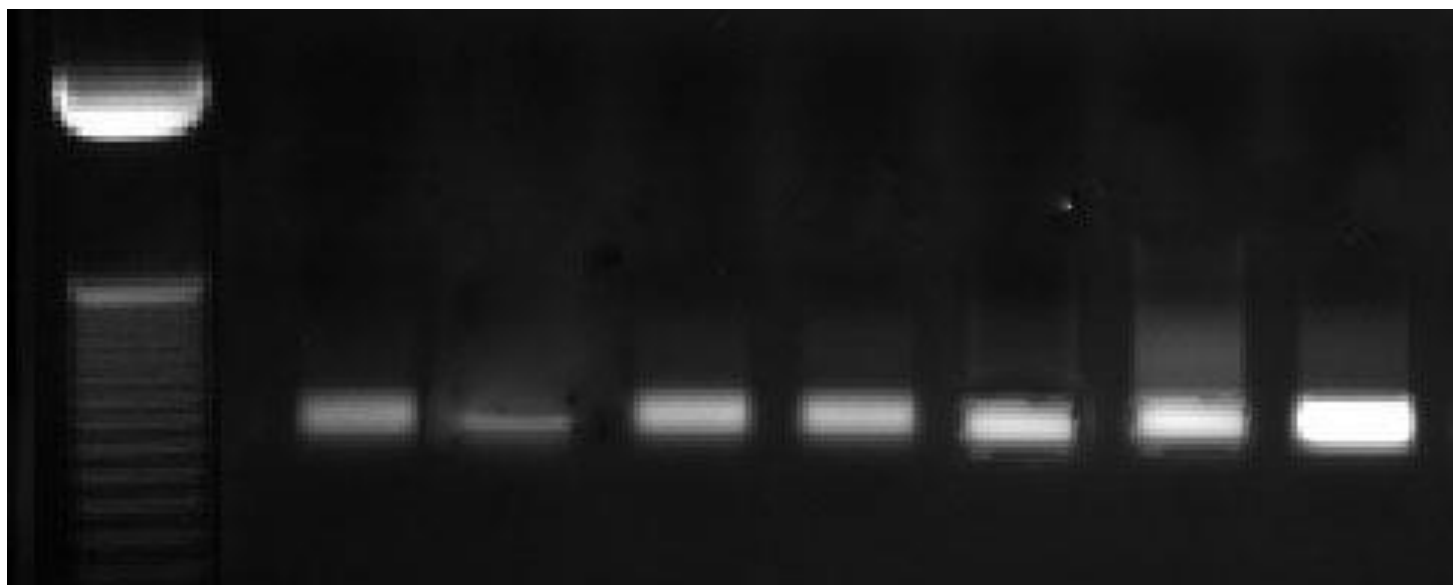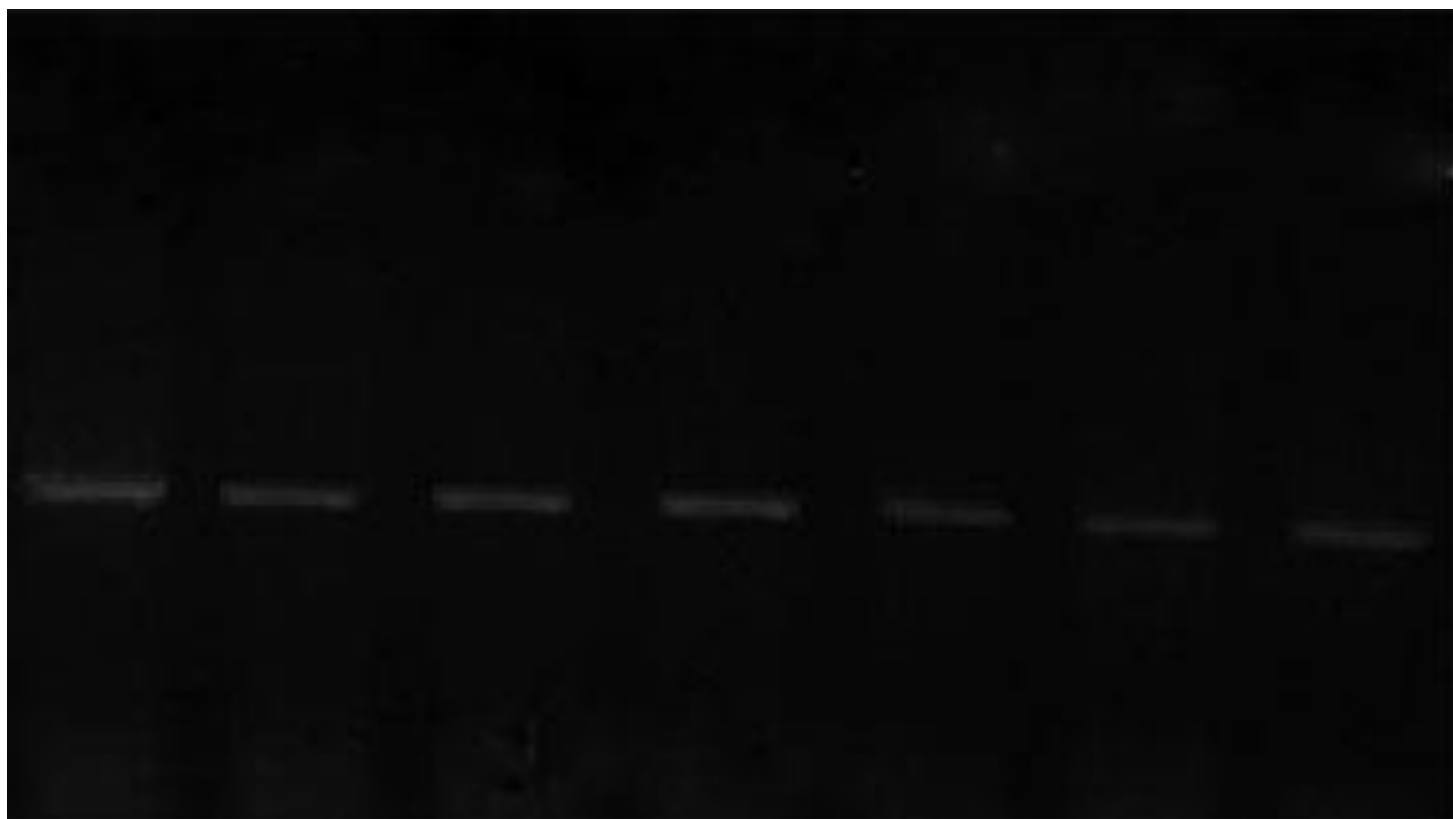

Supplement: S1 Raw images — (PDF) [file pone.0246265.s002.pdf]
